# Extracellular Vesicles-mediated recombinant IL-10 protects against ascending infection-associated preterm birth by reducing fetal inflammatory response

**DOI:** 10.3389/fimmu.2023.1196453

**Published:** 2023-08-04

**Authors:** Ananth Kumar Kammala, Angela Mosebarger, Enkhtuya Radnaa, Emma Rowlinson, Natasha Vora, Stephen J. Fortunato, Surendra Sharma, Melody Safarzadeh, Ramkumar Menon

**Affiliations:** ^1^ Division of Basic Science and Translational Research, Department of Obstetrics & Gynecology, The University of Texas Medical Branch at Galveston, Galveston, TX, United States; ^2^ Division of Maternal-Fetal Medicine, Department of Obstetrics and Gynecology, Wexner Medical Center, The Ohio State University, Columbus, OH, United States; ^3^ Department of Pediatrics, Women & Infants Hospital of Rhode Island, Providence, RI, United States

**Keywords:** pregnancy, extracellular vesicles, ascending infection, fetal membranes, anti-inflammatory, antibiotics

## Abstract

**Background:**

Fetal inflammatory response mediated by the influx of immune cells and activation of pro-inflammatory transcription factor NF-κB in feto-maternal uterine tissues is the major determinant of infection-associated preterm birth (PTB, live births < 37 weeks of gestation).

**Objective:**

To reduce the incidence of PTB by minimizing inflammation, extracellular vesicles (EVs) were electroporetically engineered to contain anti-inflammatory cytokine interleukin (IL)-10 (eIL-10), and their efficacy was tested in an ascending model of infection (vaginal administration of E. *coli*) induced PTB in mouse models.

**Study design:**

EVs (size: 30-170 nm) derived from HEK293T cells were electroporated with recombinant IL-10 at 500 volts and 125 Ω, and 6 pulses to generate eIL-10. eIL-10 structural characters (electron microscopy, nanoparticle tracking analysis, ExoView [size and cargo content] and functional properties (co-treatment of macrophage cells with LPS and eIL-10) were assessed. To test efficacy, CD1 mice were vaginally inoculated with E. *coli* (10^10^CFU) and subsequently treated with either PBS, eIL-10 (500ng) or Gentamicin (10mg/kg) or a combination of eIL-10+gentamicin. Fetal inflammatory response in maternal and fetal tissues after the infection or treatment were conducted by suspension Cytometer Time of Flight (CyTOF) using a transgenic mouse model that express red fluorescent TdTomato (mT+) in fetal cells.

**Results:**

Engineered EVs were structurally and functionally stable and showed reduced proinflammatory cytokine production from LPS challenged macrophage cells *in vitro*. Maternal administration of eIL-10 (10 µg/kg body weight) crossed feto-maternal barriers to delay E. *coli*-induced PTB to deliver live pups at term. Delay in PTB was associated with reduced feto-maternal uterine inflammation (immune cell infiltration and histologic chorioamnionitis, NF-κB activation, and proinflammatory cytokine production).

**Conclusions:**

eIL-10 administration was safe, stable, specific, delayed PTB by over 72 hrs and delivered live pups. The delivery of drugs using EVs overcomes the limitations of in-utero fetal interventions. Protecting IL-10 in EVs eliminates the need for the amniotic administration of recombinant IL-10 for its efficacy.

## 1 Introduction

The highly choreographed homeostatic state of endocrine, paracrine, and immune mediators at the fetal-maternal interface maintains pregnancy and supports the growth and development of the fetus (1). The disruption of this delicate balance in any of these systems can lead to adverse pregnancy events such as preterm birth (PTB; <37 weeks of gestation), effecting 12% of all pregnant subjects worldwide (2). PTB is a major clinical dilemma, as the contributing factors are unknown (3). A detailed clinical evaluation, however, strongly suggests that ascending infections are associated with ~ 60% of all spontaneous PTBs (4). Ascending infections leading to microbial colonization in the amniotic cavity and subsequent fetal inflammatory response are major mechanistic mediators of spontaneous PTB (5). Fetal inflammatory response and the propagation of inflammatory mediators towards uterine-fetal tissues can offset all homeostatic balances and induce unscheduled preterm labor and delivery (6). Current strategies to prolong gestation by minimizing uterine contractility and inflammation contributing to cervical remodeling on the maternal side have not reduced the incidence of PTB, warranting better strategies that can reduce fetal and maternal inflammatory activation (7–9). Although maternal inflammation has been targeted in recent studies to prolong gestation without consequential preterm birth preventive effects, reducing fetal inflammatory response has escaped serious scrutiny. Since ascending infections associated with 60% of PTB cases cause a fetal inflammatory response, using this pathway should be considered one of the strategies to mitigate PTB.

The fetal inflammatory response is pathologically diagnosed as histologic chorioamnionitis (HCA) or neutrophil infiltration of the amniochorionic membranes or fetal membranes (FMs) (10–12), which is the innermost lining of the amniotic cavity and surrounds the fetus and IL-6 cytokine levels in the cord plasma (13). HCA contributes to the PTB pathophysiology, with severe neonatal morbidity in preterm neonates (14, 15). Intervention to address fetal inflammatory response is difficult, as the kinetics of infection and its clinical manifestations are difficult to predict in a pregnant subject (14, 16, 17). Mechanistically, inflammation mediated by the master transcriptional factor NF-κB activation has been seen in most of the feto-maternal uterine tissues in settings of infection and PTB (18–22). The activation of NF-κB leads to the programming of intrauterine proinflammatory milieu, and chemotaxis of immune cells. Several compounds have been tested to reduce the activation of NF-κB, either directly or indirectly, in disparate *in vivo* animal models (23–30). Although successful in preclinical trials, none of these compounds have advanced to human clinical trials. There are multiple reasons for the lack of success expected from these preclinical trials, or hindering PTB drug development (1): inability of drugs to cross feto-maternal interface barriers to treat both the mother and the fetus, (2) drug transport studies are restricted primarily to placental perfusion models, and the lack of consideration of FM/decidual interface as a gateway for drug transport has created ambiguity and the non-reproducibility of data in *in vivo* models, and (3) teratogenicity and or the cytotoxicity of compounds that are successful in *in vitro* models.

In order to overcome these limitations, we have previously reported the use of extracellular vesicles (EVs, defined in this manuscript as 30-160 nm particles) derived from human cells as vehicles to deliver NF-κB inhibitor drugs (31). As EVs can protect the biological materials intact in the lumen, and cross placental and FM barriers, these small cellular particles were tested as a drug delivery vehicle. EVs are noncytotoxic and immune privileged (32, 33), and they exhibit organotropism, cross the placenta and FMs to reach the amniotic cavity, and have no immunogenic profile (34, 35). Our previous study used EVs engineered to contain a mutant form of IκB (super repressor [SR] of NF-κB) during their biogenesis stage (31). SR harbours phosphorylation sites that prevent NF-κB activation in response to a stimulant and keep the IκB/NF-κB complex intact and inactive in the cytoplasm, preventing NF-κB-mediated transcriptional regulation. SR has shown its efficacy in delaying intraperitoneally injected lipopolysaccharide (LPS)-induced PTB in mouse models of pregnancy (31). Its effectiveness in ascending infection, as seen in human PTB, is not clear, however, and any long-term immune suppressive properties of SR in the neonate, and/or the mother, in the mouse models are currently untested.

IL-10, a potent anti-inflammatory cytokine, has been shown to be a pregnancy compatible immune effector (36). Previous studies have shown that IL-10 has reduced the experimental fetal growth restriction and demise (37), protection against LPS induced abortion and fetal growth restriction in mice (36, 38) and reduced uterine contraction induced by the IL-1β (39). The question arises as to whether such potent biologicals will target fetal inflammatory responses more efficaciously. To address this, we electroporetically packaged IL-10 as a drug in HEK293T cell-derived EVs, now called eIL-10. IL-10 was chosen based on reports that recombinant IL-10 (rIL-10) can reduce the risk of inflammation-associated PTB (40–43). A deficiency of IL-10 in the amniotic fluid of women with amniotic infection has been reported as a factor associated with fetal inflammation and PTB (44) in animal models (44–50) as well as in *in vivo* nonhuman primate and mouse models (38, 39, 51). IL-10 inhibits the activation of NF-κB in a cell and stimulant-dependent manner. The most commonly reported mechanism of IL-10 is *via* inhibiting IκB kinase phosphorylation that prevents activation of NF-κB (52–56). By administering eIL-10, we protect the cytokine from its short half-life in the circulation, allowing its passage to the fetus so as to overcome the limitations of prior studies which showed that rIL-10 requires administration into the amniotic cavity for its success in reducing PTB (39).

In this study, we combined exosome-based drug delivery and eIL-10’s ability to control unscheduled inflammation and ascending infection-associated PTB. To test the impact of eIL-10 in reducing the fetal inflammatory response and its influx into maternal uterine tissues, we used a transgenic mouse model expressing a membrane-targeted, 2-colour fluorescent Cre-reporter allele where membrane-expressed tandem dimer Tomato (tdTomato—mT+) was expressed in all cells and tissues (57). mT+ males were mated with female wild-type (WT) mice in order to have all fetal tissues express the mT/mG construct expressing mT+, keeping maternal tissues negative. This model produces fetal cells tagged with mT+, allowing us to identify and sort fetal-specific cells from a pool of feto-maternal cells. We report that eIL-10 delayed PTB induced by ascending E. *coli* infection, resulting in the animals delivering 75% live pups. The uptake of eIL-10 uptake was seen in various feto-placental cells. Mechanistically, eIL-10 inhibited NF-κB activation in both feto-maternal tissues, and reduced fetal inflammatory response mediated by infiltrating immune cells. Follow-up experiments suggested that mothers and their offspring showed no long-term immune suppression.

## 2 Materials and methods

### 2.1 Culturing of HEK cells and media collection for isolation of EVs

HEK-293T human embryonic kidney cells were obtained from the American Type Culture Collection (Manassas, VA) and were cultured in DMEM supplemented with 10% fetal bovine serum and Pen/Strep. Cells were cultured in the T-175 flask with EVs free FBS media prepared by the 16hrs ultracentrifugation of FBS. Media will be collected for every 48 hours consistently from the cells until cells get 90% confluent. Collected media was stored at -80°C freezer for EVs isolation.

### 2.2 HEK-derived EV isolation and purification

EVs were isolated and purified with a defined centrifugation process as described previously (58). Briefly, frozen media was thawed overnight at 4°C, and sequentially centrifuged at 300 g for 10 min, 2000 g for 20 min at 4°C. Supernatants were concentrated in the Amicon^®^ ultra-15 centrifugal tube of 100 000 nominal molecular weight limit (NMWL) for 30 min at 4000 g. Concentrated media were collected, and centrifuged at 10 000 g for 30 min at 4°C. Supernatants were filtered through a 0.2 μm Nalgene™ syringe filter (Thermo Scientific, Waltham, MA, USA), and ultracentrifuged at 100,000 g in a type 70.1 Ti rotor (Beckman Coulter, Brea, CA, USA) for 2 h at 4°C. Supernatants were discarded and pellets were resuspended in ice-cold PBS and centrifuged at same speed for another 1 h to clean the EVs. Pellets were then resuspended in the PBS, aliquoted and stored at −80°C for further use. EVs free fetal bovine serum (FBS, Sigma Aldrich) were used in all EVs collection experiments (59).

### 2.3 Electroporation for loading rIL-10 into the EVs

A total of 10^9^ HEK-derived EVs and 1 μg of human r Interleukin (IL) 10 (rIL-10, Sino Biologics In US, Pennsylvania, USA) were mixed in 400 μl of electroporation buffer (1.15 mM potassium phosphate, pH 7.2, 25 mM potassium chloride, 21% Optiprep), and were electroporated using a Gene Pulser Xcell Electroporation System (Bio-Rad, Hercules, CA, USA) as previously described (58). Briefly, the mixtures were transferred in a single 4 mm cuvette and electroporated at 500 V, 125 μF, and ∞ ohms with 6 pulses, and immediately transferred to ice. After electroporation, the EVs were kept on ice and washed twice with ice-cold PBS to remove excess rIL-10 and electroporation buffer using an Amicon^®^ ultra-15 centrifugal tube (NMWL 100 000) at 1000g for 10 min. After washing, collected EVs were pooled, aliquoted and stored at −80°C for further analysis. As control, 10^9^ EVs from the same batch were electroporated using the same conditions with PBS (endotoxin-free Dulbecco’s PBS (1×), w/o Ca++ & Mg++, MilliporeSigma, Burlington, MA, USA) instead of rIL-10.

### 2.4 EV characterization

#### 2.4.1 Exosome size distribution and concentration measurements

Nanoparticle tracking analysis (NTA) was performed with Nano Sight NS300 (Malvern Panalytical Ltd., Malvern, UK) to measure the sizes and concentrations of the EVs as described previously (31, 58). The analysis settings were optimized on the day of the experiment and remained constant between the samples. EVs were diluted in filtered ultrapure water (ELGA, Bucks Marlow, UK) before running through the instrument.

#### 2.4.2 Cryo-electron microscopy

To determine the shape of EVs, 3 µL of prepared EVs suspension was pipetted onto a copper grid with quantfoil support film (QUANTFOIL, Germany). The support film was patterned with a regular array of circular holes. When the excess liquid was blotted away from the grid, a 60–120 nm-thick film of sample suspension remained in these holes. The grid was then plunged into a small crucible of liquid ethane that was cooled to its melting point by liquid nitrogen. In the liquid ethane, the sample suspension was cooled at over 10,000 degrees/s, solidifying the water in an amorphous state. This “vitrification” process preserves the EVs in their native state without distorting their geometry by crystallization and density change. The vitrified sample on the grid was then placed in a Gatan 626 specimen holder (Gatan, Pleasanton, CA), which was placed in a JEOL 2100 TEM (JEOL, Osaka, Japan). The samples were imaged with a 200 kV electron beam from a LaB6 emission source, and images were recorded on a Gatan US4000 CCD camera. Images were captured at 15,000–30,000 magnification.

#### 2.4.3 Western blot for the analysis of IL-10 and exosomal markers

EVs were lysed and analysed as described previously with some modifications (31, 58). Briefly, the lysates were mixed with 4× loading buffer (Bio-Rad) in non-reducing conditions and without heating. 50 μl lysates were loaded into each well of 4–15% gradient polyacrylamide gels (Bio-Rad) and subjected to electrophoresis. Subsequently, the proteins were transferred to polyvinylidene fluoride (PVDF) membranes (Bio-Rad) by semi-dry electrophoretic transfer (Bio-Rad), and the membranes were blocked in 5% nonfat dry milk/TBST (Tris-buffered saline, 0.1% Tween20) for 2 h at room temperature (RT). The membranes were then incubated with primary antibodies of CD63 (Novus Biologicals, Centennial, CO, USA, Clone: MX-49.129.5, [NBP2- 32830]) and CD81 (Cell Signalling Technology, Cat. No.: MAB6435, Lot. No.: 531413) diluted at 1: 400 in 5% nonfat dry milk/TBST overnight at 4°C, with secondary antibodies (SouthernBiotech, Birmingham, AL, USA, Cat. No.: 1030-05, Lot: K3515-T566, DF: 1: 15 000) for 1 h at RT. Protein bands were visualized using an enhanced chemiluminescent western blotting solution (Bio-Rad) with ChemiDoc™ Imaging System (Bio-Rad).

#### 2.4.4 IL-10 concentration measurement in the EVs

IL-10 concentrations in electroporated EVs were measured with ELISA (BD Biosciences, USA). Electroporated control (eCTRL) and IL-10 EVs (eIL-10) were lysed with a 10× radioimmunoprecipitation assay (RIPA) lysis buffer (0.5 M Tris, pH 8.0; 1.50 M NaCl: 10% (v/v) Triton X-100; 10 mM EDTA, pH 8.0; and 10% (w/v) sodium dodecyl sulphate-SDS) supplemented with protease (MilliporeSigma), phosphatase (Thermo Scientific) inhibitor cocktails, and phenylmethylsulfonyl fluoride (PMSF, Honeywell Fluka, Charlotte, USA) for 5 min at RT while vortexing intermittently. IL-10 ELISA was performed according to the manufacturer’s instruction and colour development was measured with a Synergy™ H4 plate reader (BioTek™, Winooski, VT, USA). The results of the ELISA were used to determine the amount of IL-10 (ng) per exosome.

#### 2.4.5 ExoView detection of IL-10

To validate and determine the loading efficiency of the electroporation methods, EVs were analysed using the ExoView platform (NanoView, Boston, MA, USA) following the manufacturer’s procedure with modifications. ExoView allows the detection of specific cargo protein at a single-vesicle level (58, 60). Briefly, 35 μl of EVs (1 × 10^9^/ml) from eCTRL and eIL-10 were diluted in solution A (NanoView Biosciences) and incubated on tetraspanin microarray chips placed in a 24-well plate overnight at RT. Each chip was pre-coated with CD9, CD63, CD81 antibodies and MIgG control antibodies. Solutions and buffers provided by the manufacturer for ExoView experiments are proprietary to the company and their exact composition is not known to these investigators. The following day, unbound EVs were washed 3 times for 3 min in a 500 rpm shaker in solution A. EVs bound to the capture spots were then fixed and permeabilized with the ExoView cargo kit according to the manufacturer’s protocol. Briefly the bound EVs were fixed with solution C (NanoView Biosciences) for 10 min, washed as previously described, and lysed in solution D (NanoView Biosciences) for 10 min and washed again as described. IL-10 primary antibody (Cell Signaling Technology, Danvers, MA, USA) was labelled with Alexa Fluor™ 555 labelling kit (Invitrogen) according to the manufacturer’s instructions. Briefly, 1 μg IL-10 antibody was labelled with 5 μl of Alexa Fluor™ 555 for 5 min at RT, followed by quenching with 5 μl of blocking reagent (Invitrogen) and used immediately after the labelling. EVs were then co-stained with AF555 conjugated IL-10 antibody (Cell Signalling Technology) (0.3 μg per chip), CD9 and CD63 antibodies diluted in blocking solution (NanoView Biosciences) for 1 h at RT in the dark. The tetraspanin microarray chips were then sequentially washed 3 times for 5 min in solution A, and in solution B (NanoView Biosciences), and 5 times for 5 min in Milli-Q water (ELGA) at 500 rpm shaker. The chips containing EVs were then carefully dried from the final water wash and placed on absorbent paper and then imaged on the ExoView R100 instrument (NanoView Biosciences) using the nScan 2.9.3 acquisition software. The size distribution, concentration, and IL-10 loading efficiency were calculated using NanoViewer 2.9.3 provided by NanoView Bioscience, and output was displayed and stored in an Excel spreadsheet. To ensure the incorporation of rIL-10 FITC inside the lumen of EVs rather than surface binding, we have treated EVs with proteinase K to shave membrane bound proteins and washed in amicon tubes for Exoview analysis.

#### 2.4.6 HEK reporter cell assay

HEK Blue IL-10 reporter cell lines were incubated with eIL-10 at different doses to determine the functional activity of eIL-10. Naïve EVs were used as controls. All cell lines contained an IL-10R-inducible secreted embryonic alkaline phosphatase (SEAP) reporter gene. Activation of IL-10 signaling leads to the expression of SEAP, which can be detected in culture supernatants upon addition of the substrate Quanti-Blue (InvivoGen). The HEK-Blue cells were cultured as per kit manufacturing protocol. In short, HEK-Blue cells were stimulated in a volume of 200 μl in 96-wells plates for 24 hours according to the manufacturer’s instructions. An equivalent dose of rIL-10 (100ng) to eIL-10 was added to the wells and incubated for 2 hours. rIL-10 was considered the respective positive control and Naïve EVs at equal concentrations of eIL-10 EVs considered as negative control.

#### 2.4.7 Cell cycle analysis of eIL-10 using flow cytometry

Naïve EVs and eIL-10 were used to treat RAW 264.7 cells for 24 hours. Cells were harvested after media collection using trypsin EDTA (Corning, Corning, NY) and centrifuged for 10 minutes at 3000 RPM. The supernatant was removed, and cells were resuspended in 50 μL PBS. Cell cycle analysis was performed using the Coulter DNA Prep Reagents Kit (Beckman Coulter, Indianapolis, IN). Briefly, 50 μL of DNA Prep LPR was added to each sample and vortexed. Then 1.0 mL DNA Prep Stain was added to the tubes, vortexed and run immediately on the Cytoflex flow cytometer (Beckman Coulter). After selecting for single cells, gating was set for the control cells and applied to histograms for the EVs-treated RAW cells using Cytexpert (Beckman Coulter).

#### 2.4.8 Cytotoxicity assay

In order to determine the cytotoxicity of eIL-10, Raw 264.7 cells were stained using the Dead Cell Apoptosis Kit with Annexin V Alexa Fluor 488 & PI (Life Technologies, Carlsbad, CA) as reported previously (61). Briefly, cells were harvested after 48 h of eIL-10 (500ng) by trypsinization and centrifuged for 5 min at 3000 × g. Cell pellets were washed with cold 1× PBS and centrifuged at 3000 × g for 5 min. Pellets were resuspended in 100 μL 1× annexin binding buffer supplemented with 5 μL Alexa Fluor 488 Annexin V and 1 μL 100 μg/mL propidium iodide (PI). After a 15 min incubation, 400 μL annexin binding buffer was added, and samples were run immediately on the CytoFlex flow cytometer (Beckman Coulter). Unstained RAW264.7 cells were used as negative controls for gating. RAW264.7 cells treated with 100ng/mL of LPS were considered as positive controls. Data were analysed using CytExpert software (Beckman Coulter).

#### 2.4.9 Enzyme-linked immunosorbent assay for IL-6, TNFα and IL-1 beta

Culture media collected from RAW 264.7 mouse macrophages 6 h post-treatment of either Naïve EVs, LPS (100ng/mL), LPS+eIL-10(500ng) were tested for mouse IL-6, TNFα and IL-1β using the Mouse IL-6 Quantikine ELISA (M6000B, Minneapolis, MN, USA) Mouse TNF ELISA Set (555268, BD Biosciences, San Jose, CA) and Mouse IL-1β ELISA Set (561667, BD Biosciences, San Jose, CA) respectively. Standard curves were developed using duplicate samples of known-quantity r proteins that were provided by the manufacturer. Sample concentrations were determined by relating the absorbance of the samples to the standard curve using linear regression analysis.

#### 2.4.10 Animal care

All animal procedures were approved by the Institutional Animal Care and Use Committee (IACUC) at the University of Texas Medical Branch, Galveston with approved protocol number 041107F. Timed-pregnant CD-1 mice (Charles River Laboratories, Houston, TX) were received on gestational day (E) 14. TdTomato mice were breed in-house and mated for timed pregnancy with C57BL/6J female mice. Mice were housed in a temperature- and humidity-controlled facility with automatically controlled 12:12-h light and dark cycles with regular chow and drinking solution provided ad libitum. Certified personnel and veterinary staff provided regular maintenance and animal care according to IACUC guidelines. Prior to tissue collection, animals were euthanized by CO_2_ inhalation according to the IACUC and American Veterinary Medical Association guidelines.

#### 2.4.11 Bio-availability of eIL-10 at placenta and FM tissues

To assess the *in vivo* tissue bio-availability of eIL-10, rIL-10 was conjugated with FITC as described in manufacturing kit protocol (3326-32-7, Sigma, MO, USA). Briefly, 2mg/mL of rIL-10 was prepared in 0.1 M sodium carbonate buffer, pH 9. 50uL of FITC solution was added to 1 ml of protein solution slowly and stirred continuously. The mixture of FITC and protein was incubated in the dark for 8 hours at 4°C. NH_4_Cl was added to final concentration of 50mM and incubated for 2 hours at 4°C to quench the reaction. The mixture was washed with ice-cold PBS twice using Amicon 10K tubes by centrifugation at 1000g for 10 min for each wash to remove unbound FITC. Flowthrough was discarded and the conjugated protein was quantified by ELISA. 500ng of EVs loaded with FITC IL-10 and unpacked FITC rIL-10 was injected intravenously into pregnant CD-1 on day E-15. After 3 hours of injection, mice were euthanized. Placenta and fetal membrane tissues were excised and imaged using the IVIS Spectrum imaging system (PerkinElmer, Waltham, MA, USA) at UTMB Biomedical Imaging facility. The image was processed using Living Image software (IVIS Imaging systems). The experimental design is depicted in **Supplementary Figure 2B**.

#### 2.4.12 LPS-induced PTB and eIL-10 treatment

On gestational day E15 (equivalent to ~75% completed gestation in mice or ~28 weeks in humans), pregnant dams were intraperitoneally injected with one of the following: PBS or LPS {serotype 055:B5, Sigma-Aldrich, St. Louis, MO [100 µg for CD-1 mice]. Thirty minutes after injection, animals were intravenously injected with either PBS, eIL-10 (500ng), Naïve EVs (Equivalent particles of 500ng of eIL-10), recombinant IL-10 (500ng). Treatments were repeated every 2 hours for a total of three injections (CD-1). Animals were monitored for preterm delivery, which was defined as the delivery of at least one pup on or before E18.5, using Harvard Bioscience monitoring systems. A subset of CD-1 mice (n=5) was euthanized at the time of LPS-induced preterm labour (12 hours after injection), and FMs and plasma was collected for signaling and cytokine analysis.

#### 2.4.13 E. coli induced ascending infection model of PTB and eIL-10 treatment

The detailed protocol for E. *coli* induced ascending infection has been described previously (62). Briefly, on gestational day E-15, CD-1 mice were vaginally administered with 10^10^ CFU E. *coli (*ATCC 12014 Escherichia coli O55:K59(B5):H., Thermo Fisher Scientific, Remel Products, Lenexa, KS, USA, Lot# 496291). After two hours of E. *coli* administration mice were treated with either PBS, eIL-10 (500ng) or Gentamicin (10mg/kg). PBS/eIL-10 injected were repeated every 2 hours for a total of three injections, whereas one gentamicin injection was given injection after 4 hours. The dosing schedule of the PBS/eIL-10 was determined based on studies using NF-κB inhibitors in the extracellular vesicles (31) and amniotic recombinant IL-10 studies in delaying preterm delivery (39). Gentamicin dose and schedule was determined based on our preliminary studies with different doses (10, 20 and 64mg/Kg) and at different frequencies (1,2 and 3 doses). Animals were monitored for preterm delivery as mentioned above. A subset of CD-1 mice was euthanized at the time of E. *coli* -induced preterm labour (24 hours after injection), and FMs and plasma was collected for signaling and cytokine analysis.

#### 2.4.14 LPS induced immune challenge assay

Live pups from the infection-induced model treated with liquid broth, eIL-10 and Gentamicin treated groups were weaned after four weeks. After six weeks of age, pups were subjected to Immune challenge assay induced by LPS at dose of 100ug intraperitoneally. Mice were euthanized after 2 hours of the treatment and blood was collected in a heparinized tubes to analyse proinflammatory cytokines IL-6 and TNF-alpha by ELISA as mentioned above.

#### 2.4.15 Determination of signaling pathway of eIL-10 in mouse macrophages

The molecular pathway of eIL-10 was determined using RAW264.7 Mouse macrophage cell lines. AW 264.7 cells (murine macrophage cell line) were purchased form American Type Tissue Culture Collection (ATCC, Rockville, MD). RAW 264.7 cells were suspended in complete medium; Dulbecco’s modified Eagle’s medium (DMEM) supplemented with 10% fetal bovine serum, 100 U/mL penicillin, and 100 U/mL streptomycin. Cells were plated in 24-well plates at a density of 5 × 105 cells/well. All experiments were performed in a humidified atmosphere under 5% CO2 at 37°C. RAW 264.7 cells were harvested in ice cold PBS after stimulation with either PBS, LPS, LPS+eIL-10 and LPS+rIL-10 from 0 to 30 mins. Cells were then lysed in 70 μl of buffer A (10 mM HEPES pH 7.9, 1.5 mM MgCl2, 10 mM KCl, 0.25% v/v NP-40, 0.5 mM dithiothreitol (DTT), 0.5 mM phenylmethylsulfonyl fluoride (PMSF) in de-ionized water (dH2O) for 20 min on ice, to yield the cytoplasmic cellular fraction. Further protein lysate was prepared as mentioned above to run western blotting. The membranes were incubated with primary antibodies pNF-κB (ab32536, ABCAM, MA, USA), IKB-alpha (ab32518, ABCAM, MA, USA), pSTAT3 (9131S, Cell Signaling, MA, USA), SOCS3 (ab280884, ABCAM, MA, USA), pERK(4370, Cell Signaling, MA, USA), p44/42 MAPK (4695, Cell Signalling, MA, USA) at a dilution of 1:1000 in EveryBlot Blocking buffer (BioRad) and beta actin (ab6276, ABCAM, MA, USA) 1:10000 for overnight shaking at 4°C temperature. The fluorescence secondary antibodies used were Goat anti-Rabbit IgG H & L (IRDye 800CW) (ab216773, ABCAM, MA, USA) and Goat anti-mouse IgG H & L (IR Dye 680RD) (ab216776, ABCAM, MA, USA) at a dilution of 1:15000 at RT for one hour. Protein bands were visualized using ChemiDoc™ Imaging System (Bio-Rad).

#### 2.4.16 Immunocytochemistry staining to determine inhibition of nuclear translocation of NF-κB by eIL10

RAW 264.7 cells were plated at 2.3x10^4 cells/well in an 8 well plate, with a volume of 300μl media/well. They were allowed to grow overnight. Media was then removed from the cells and treatment was administered in 300μl solutions in media. Cells were treated with media as a control, 500ng/mL of LPS, 500ng/mL of r IL10 (rIL10), 500ng/mL of eIL10, co-administration of eIL10 and LPS, or co-administration of rIL10 and LPS. Treatment lasted 30 minutes incubated at 37°C. The reaction was then stopped by adding cold PBS to each well. Cells were fixed with 4% paraformaldehyde for 20 min, then permeabilized with Triton. They were then stained with phospho-NF-κB Ser536 Rabbit mAb (Cell Signaling Technology, 3033) at 1:1000 in BSA overnight, then with Goat Anti-Rabbit Alexa Fluor 488 (Abcam, ab150077) in 1:1000 BSA for one hour, and DAPI (Invitrogen, R37606) for five minutes. Slides were mounted and allowed to dry overnight, and images were then captured using a Keyence microscope.

#### 2.4.17 Immunohistochemistrystaining of FM tissue of neutrophil infiltration

Paraffin-embedded sections were cut at 5 μm thickness, mounted on pre-coated hydrophilic glass slides (Matsunami Glass, Bellingham, WA), dried at 37°C to ensure adherence to the slides, and stored at 4°C until use. Sections were baked at 50°C overnight before staining. Paraffin sections were deparaffinized in 3 changes of xylene for 10 min each, then rehydrated through a series of graded alcohols with a final rinse in distilled water. Sections were then subjected to antigen retrieval by heating at 121°C in citrate buffer for 20 min. The slides were then rinsed in distilled water, TBS, and blocked with 3% BSA/TBS-T for 1 h at RT. Tissues were then stained with anti-Ly-6G/Ly-6C antibody (RB6-8C5) (Abcam, Cat#ab25377 Lot : GR3441657- with 1:200) diluted in 3% BSA/TBS-T overnight at 4°C. The next day, the tissues were washed then incubated with secondary antibody (Alexa 488, Abcam, Cat# ab150157 Lot: GR3444870-1 with 1:1000) for 2 h at RT followed by DAPI staining. Images were obtained and analysed as described above using the BZ-X800 Analyser (Keyence Corp).

#### 2.4.18 High dimensional single cell profiling of FM tissues by mass cytometry

##### 2.4.18.1 Preparation of single cell suspensions of the murine tissues for CyTOF

Murine tissues (fetal membranes and placenta) collected after experiment were digested with 1mL of Accutase (Innovative cell technologies) for 30 minutes at 37°C with gentle shaking. The tissues were transferred into a C-tube (Miltenyi Biotec) H-cord-01 setting. The tissue was washed with 20mL of 1x PBS, filtered through a 40-µm, and centrifuged at 1250g for 10 minutes at 4°C. Cell pellets were incubated in 5mL of red blood cells (RBC) lysis buffer (biolegend) for 10 minutes at room temperature. Cells were centrifuges at 1250g for 10 minutes at 22°C. The cell pellets were resuspended in the Maxpar staining buffer for further analysis.

##### 2.4.18.2 Antibodies

The CyTOF panel was designed based on the high-throughput screening results, as well as including proteins known to regulate myeloid cell functions (such as MerTK and Axl), transcription factors and signaling molecules known to be relevant to inflammation during pregnancy (NF-κB and MAPK pathway). A summary of antibodies used for each panel can be found in **Supplementary Table 2**. The antibodies were sourced from the MD Anderson Cancer research centre flow core facilities. (MDACC, Texas, Houston), or custom conjugated using the Maxpar antibody conjugation kit (Fluidigm, Markham, ON, Canada) following the manufacturer’s protocol. After being labelled with their corresponding metal conjugate, the percentage yield was determined by measuring their absorbance at 280 nm using a Nanodrop 2000 spectrophotometer (Thermo Scientific, Wilmington, DE). Antibodies were diluted using Candor phosphate-buffered saline (PBS) antibody stabilization solution (Candor Bioscience GmbH, Wangen, Germany) to 0.3 mg/mL and then stored at 4°C.

##### 2.4.18.3 Antibody staining

Single cell suspension samples were resuspended in Maxpar staining buffer for 10 min at room temperature on a shaker to block Fc receptors. Cells were mixed with a cocktail of metal-conjugated surface marker antibodies (**Supplementary Table 2**), yielding 500-μL final reaction volumes, and stained at room temperature for 30 min on a shaker. Following staining, cells were washed twice with PBS with 0.5% BSA and 0.02% NaN3. Next, cells were permeabilized with 4°C methanol for 10 min at 4°C. Cells were then washed twice in PBS with 0.5% BSA and 0.02% NaN3 to remove remaining methanol. They were stained with intracellular antibodies in 500 μL for 30 min at room temperature on a shaker. Samples were then washed twice in PBS with 0.5% BSA and 0.02% NaN3. Cells were incubated overnight at 4°C with 1 mL of 1:4,000 191/193Ir DNA intercalator (Standard BioTools, Inc., Markham, ON) diluted in Maxpar fix/perm overnight. The following day, cells were washed once with PBS with 0.5% BSA and 0.02% NaN3 and then twice with double-deionized water (ddH20).

##### 2.4.18.4 Mass cytometry

Prior to analysis, the stained and intercalated cell pellet was resuspended in ddH2O containing polystyrene normalization beads containing lanthanum-139, praseodymium-141, terbium-159, thulium-169 and lutetium-175 as described previously (59). Stained cells were analysed on a CyTOF 2 (Standard BioTools Inc, Markham, ON) outfitted with a Super Sampler sample introduction system (Victorian Airship & Scientific Apparatus, Alamo, CA) at an event rate of 200 to 300 cells per second. All mass cytometry files were normalized using the mass cytometry data normalization algorithm freely available for download from https://github.com/nolanlab/bead-normalization.

##### 2.4.18.5 Data Analysis

CyTOF data sets were first manually gated using the Standard BioTools/Fluidigm clean up procedure, including Gaussian discrimination (Markham, ON) in FlowJo V10 (FlowJo LLC). Each sample was then given a unique sample ID, then all samples were concatenated into a single.fcs file. This concatenated file was further analysed using T-Distributed Stochastic Neighbour Embedding (t-SNE) in FlowJo V10, using equal numbers of cells from LB, E. *coli* and E*. coli*+eIL-10 treated mouse FM, all surface markers, and the following settings: Iterations, 3000; Perplexity, 50; Eta (learning rate), 4105. Heatmaps of marker expression were generated using the Colour Map Axis function. The resulting t-SNE plot visualized as a heatmap of marker expression of immune cells. In order to explore the phenotypic diversity of immune cell populations in the different groups of mouse FM tissues, we applied a K-nearest-neighbour density-based clustering algorithm called Phenograph. This algorithm allows the unsupervised clustering analysis of data from single cells. Output is organized using the cluster explorer tool to visualize the phenotypic continuum of cell populations. This tool creates an interactive cluster profile graph and heatmap and displays the cluster populations on a tSNE plot.

## 3 Results

### 3.1 Generation of EV-enclosed IL-10 by electroporation

**Figure 1:**
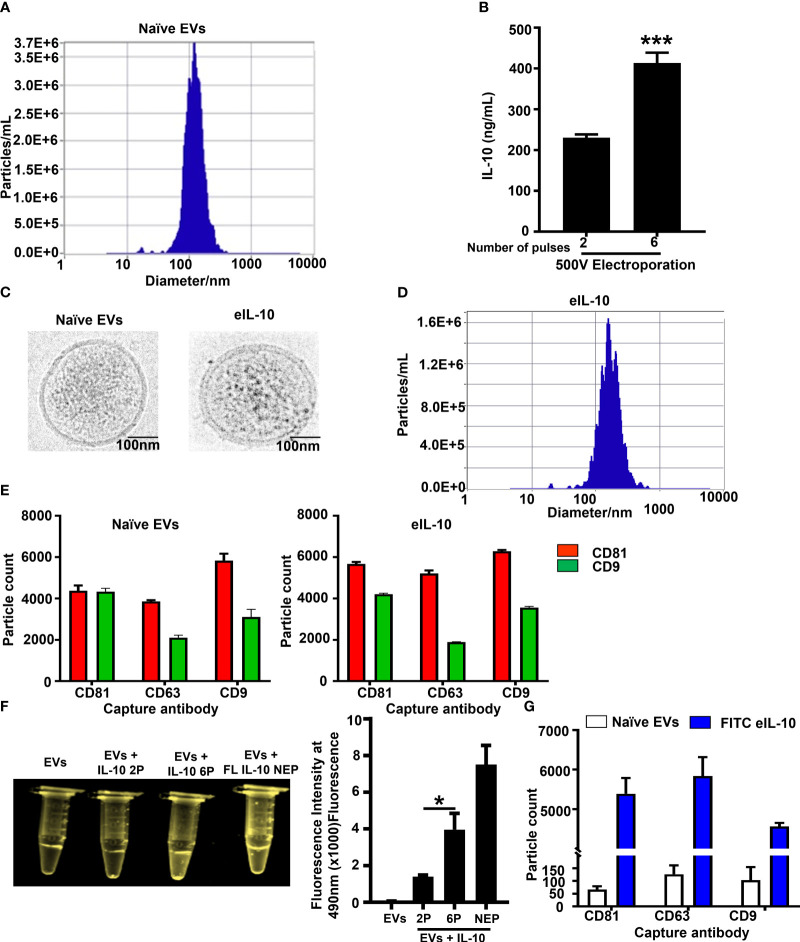
Properties of naïve EVs vs EVs encapsulating IL-10. **(A)**. Nanoparticle Tracking Analysis using ZetaView showing EV size in nanometers prior to electroporation. **(B)**. Optimization of electroporation protocol. IL-10 was encapsulated in EVs *via* sonication or electroporation at 500 Volts with either 2 or 6 pulses. Six pulses at 500 V were optimum and provided the highest loading of IL-10 within EVs. These conditions were used for the generation of eIL10 in further experiments. **(C)**. Cryo EM images showing the morphology of EVs. Representative images of naïve and electroporated EVs show similar morphology. Both EVs were round with double layered membranes. **(D)**. Nanoparticle Tracking Analysis using ZetaView showing EV size in nanometers after electroporation. The particles do not show a significant change in size after electroporation compared to nonelectroporated EVs (1A). **(E)**. ExoView analysis of EV markers: CD81 (red), CD63, and CD9 (green) were determined by ExoView and confirmed the presence of EV markers that are not altered by electroporation or encapsulation of IL-10. **(F)**. Confirmation of IL-10 loading in EVs. Recombinant (r) IL-10 was conjugated with FITC and its presence in EVs was determined by fluorescence under UV light. EVs electroporated with FITC conjugated IL-10 at 6 pulses have shown higher fluorescence intensity compared to the 2 pulses of electroporation. EVs mixed with FITC IL-10 and non-electroporated has shown higher fluorescence intensity as positive control. The fluorescence intensity was measured at 490nm using spectrophotometer. **(G)**. Confirmation of IL-10 loading in EVs using Exoview analysis. EVs captured to ExoView chips using tetraspanin markers were probed with IL-10 antibody to detect its luminal presence. Compared to naïve exosomes (white), eIL-10 EVs (blue) show presence of IL-10, and loading efficacy was calculated up to 70%. Data are shown as means ± SEM. For all groups, n ≥ 5. Data are shown as means ± SEM. Data were analyzed using a one-way ANOVA with a Tukey’s post hoc test. *P ≤ 0.05, ***P ≤ 0.001.

We have already reported that EVs are communication channels between maternal and fetal tissues (59, 63) and can be used as drug delivery vehicles. This feature of EV is utilized in our approach to deliver drugs to treat PTB-associated fetal inflammation. For the electroporation of IL-10, EVs were isolated from HEK293T cells using ultracentrifugation followed by size exclusion column purification. As shown in **Figure 1A**, EVs had an average size of 129.5 nm. We used 1 x 10^9^ EVs to load 1 μg of IL-10. The electroporation parameters for loading IL-10 were 500 volts and 125 Ω, and they were optimized with both 2 and 6 pulses. We used 6 pulses for optimum loading of IL-10 in our experiments, with maximum loading efficiency as shown in **Figure 1B**. We performed additional tests to determine whether electroporation has altered the characteristics of EVs and function of the cargo. As shown in **Figure 1C**, cryo-EM determined that the structural integrity of EVs was not disrupted by electroporation. Both naïve and electroporated EVs showed similar morphologies and lipid bilayer structure. Neither the size of the EVs (as determined by nanoparticle tracking analysis; average size – 135.6 nm) (**Figure 1D**) nor the tetraspanin markers (CD81, CD63 and CD9) as determined by ExoView analysis were altered by electroporation (**Figure 1E**). To further confirm this, the endosomal sorting complexes required for transport (ESCRT) protein expression in EVs (TSG101) were tested using western blot, as shown in **Supplementary Figure 1A**, and TSG101 was observed in both naïve and electroporated EVs.

Next, we tested the loading of IL-10 in EVs. First, we conjugated rIL-10 with FITC and electroporated it to become EVs’ cargo. The fluorescence intensity was measured at 490nm using spectrophotometer. As shown in **Figure 1F**, six pulses produced maximum efficiency of loading, and we visually confirmed the highest fluorescence under UV light. Controls (naïve EVs and EVs electroporated without rIL-10) did not show any fluorescence under UV light, confirming the luminal presence of IL-10 in the EVs. We further confirmed this with western blot (**Supplementary Figure 1B**), where IL-10 was present in electroporated EVs and electroporated naïve EVs were negative. Next, we confirmed the loading efficiency of rIL-10 in EVs using ExoView analysis. The loading efficiency was determined to be ~ 70% in EVs after electroporation (**Figure 1G**). These results confirm that we have successfully engineered EVs to contain IL-10, and the approaches used did not change the characteristics of the EVs. EVs loaded with IL-10 (eIL-10) were used in our subsequent experiments.

### 3.2 eIL-10 activates IL-10-mediated signaling pathways and inhibits inflammatory cytokine production

**Figure 2:**
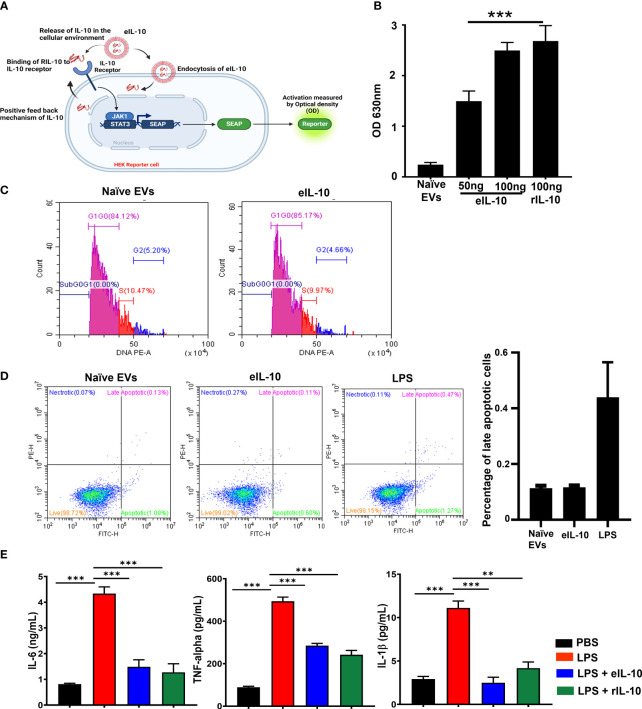
Activity of eIL10 at IL-10 pathway and cytokine production. **(A)**. Schematic of secreted embryonic alkaline phosphatase (SEAP) reporter assay to determine the functional properties of IL-10 in EVs. HEK cells transfected with SEAP reporter where IL-10/IL-10 receptor binding leads to signaling activation and causing a color change. The color change can be measured by optical density. **(B)**. Optical density (OD) measured in SEAP-transfected cells. Naïve EVs does not contain IL-10. eIL10 shows binding to the receptor in a dose-dependent manner, contributing to SEAP activation as detected by increased OD. **(C)**. Determining the impact of eIL-10 on cell cycle using flow cytometry. Cell cycle analysis was performed in RAW264.7 (mouse macrophage) cells treated with naïve EVs or eIL10. Flow cytometry showed no significant changes in the number of cells found in each stage of the cell cycle. **(D)**. Determining the impact of eIL-10 on cell fate using flow cytometry. Analysis of apoptosis and necrosis using Annexin and propidium iodide staining in RAW cells treated with naïve EVs or eIL10. LPS treated cells were considered as positive control. Neither naïve nor eIL-10 produced any necrosis of cells. Similarly, apoptotic cell deaths were lower compared to positive control group. **(E)**. Functional properties of eIL-10. ELISA was used to measure the levels of inflammatory cytokines produced by RAW264.7 cells (Mouse macrophages) treated with LPS and co-treated with either eIL10 or rIL10. LPS (red)-induced IL-6, TNF-α, and IL-1β co-treatment with eIL-10 (blue) rIL-10 (green) reduced the production of pro-inflammatory cytokines confirming functionally viable IL-10 inside the EVs. For all groups, n ≥ 5. Data are shown as means ± SEM. Data were analyzed using a one-way ANOVA with a Tukey’s *post hoc* test. **P ≤ 0.01, ***P ≤ 0.001.

After confirming that EVs remain intact and were loaded with rIL-10, we tested the functional activity of eIL-10 in HEK blue IL-10 reporter cell lines. HEK-Blue™ IL-10 cells were generated by stable transfection of the human embryonic kidney HEK293 cell line with the genes encoding hIL-10R α and β chains, human STAT3, and the STAT3-inducible secreted embryonic alkaline phosphatase (SEAP) reporter (64). As depicted in **Figure 2A**, the binding of eIL-10 to its receptor on the surface of HEK-Blue™ IL-10 cells triggers JAK1/STAT3 signaling, and the subsequent production of SEAP. eIL-10 at a dose of 50 ng and 100 ng, as well as rIL-10, have shown the activation of IL-10-mediated signaling by changing the colour of SEAP as determined by optical density (**Figure 2B**). As expected, naïve EVs remained negative, and did not show activation of the IL-10 signaling. The functional activity of the eIL-10 was also tested in mouse macrophage RAW cells to assess its effect on cell cycle progression and apoptosis. eIL-10 (500 ng) treatment of RAW cells neither stopped cell cycle progression nor caused cell death or any other adverse cell fates, as was the case with naïve EVs (**Figures 2C, D**). Previous studies have shown that rIL-10 inhibits the LPS-induced production of inflammatory cytokines in macrophages, including tumour necrosis factor α (TNF-α), IL-6, and IL-12 (48, 65, 66). Consistent with prior data, eIL-10 at a dose of 500 ng inhibited the production of inflammatory cytokines IL-6, TNF- α and IL-1β in mouse macrophages induced by LPS (100 ng/mL) (**Figure 2E**). These results confirm that eIL-10 is functionally active and elicits anti-inflammatory properties.

### 3.3 eIL-10 entails enhanced in utero bio-availability and delays PTB

**Figure 3:**
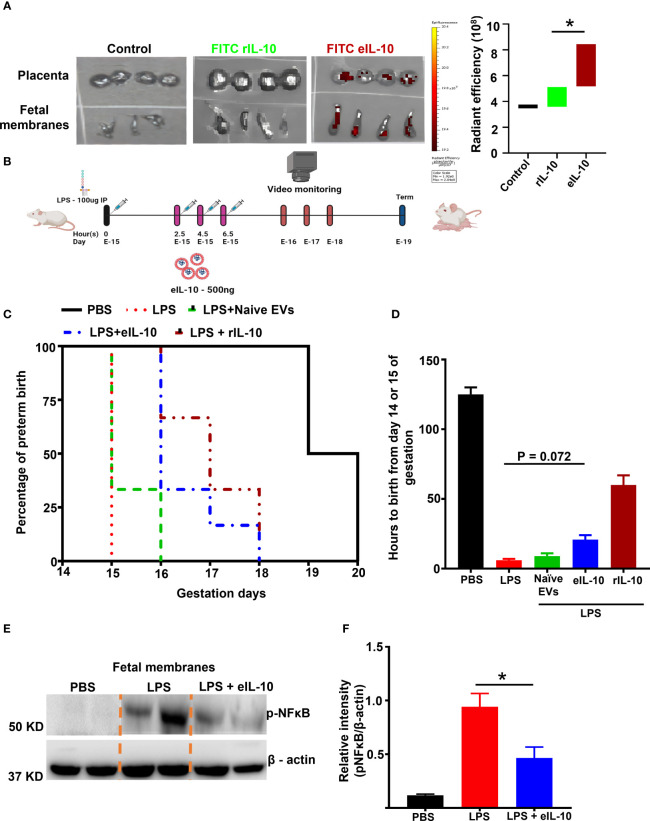
Bio-availability and effect of eIL10 in LPS-induced PTB mouse model. **(A)**. Left panel: Representative *In vivo* imaging system (IVIS) images. Fluorescently labeled IL-10 is seen in the placenta and fetal membrane when it is packaged in the EVs (FITC eIL-10) but not when rIL-10 was injected directly (FITC rIL-10). Controls remain negative, Right panel: Radiant efficiency in target tissues is shown for eIL10 (red) and rIL-10 (green). **(B)**. Schematic representation of LPS- (100µg) (intraperitoneally injected) induced PTB model and its treatment using eIL-10 (500ng) (intravenously injected through the tail vain). **(C)**. Survival curve comparing PBS (black), LPS (red), LPS + eIL-10 (blue), LPS + Naïve EVs (Green), LPS + rIL-10 (brown) treatment groups. LPS injections caused PTB within 24 hours, while LPS + eIL-10 delayed PTB by an average of 24 hours, compared to controls. For all groups, n< 8. **(D)**. Bar graph of number of hours of pregnancy maintained after eIL-10 treatment. eIL-10 delays PTB in LPS-challenged pregnant CD-1 mice. **(E)**. Representative western blot images of fetal membranes (N=5) collected 6 hours after treatment with LPS and eIL-10. eIL-10 treatment reduced NF-κB activation (reduction of Phosphorylated (p) NF-κB) compared to LPS. **(F)**. The relative levels of the P- NF-κB significantly reduced with the treatment of eIL-10 compared to LPS treatment alone in fetal membranes. For all groups, n ≥ 5. Data are shown as means ± SEM. Data were analyzed using a one-way ANOVA with a Tukey’s *post hoc* test. *P ≤ 0.05.

Enhancing the bio-availability of rIL-10 for *in vivo* function is an existing challenge (67, 68). rIL-10 was conjugated with Fluorescein isothiocyanate (FITC) to determine the bio-availability of eIL-10 in reproductive tissues. FITC labelled rIL-10 was incorporated into the EVs by electroporation, as shown in **Supplementary Figure 2A**. FITC eIL-10 was injected intravenously in healthy pregnant mice. Within 3 hours, the eIL-10 content was noticeable in the placenta and FMs compared to the control and free rIL-10 (**Figure 3A**). These data suggest that eIL-10 can cross the feto-maternal barriers and reach intrauterine tissues. Along with intrauterine uterine tissues, we have also evaluated the eIL-10 tropism in other tissues and observed that Lungs, Liver and Uterus has also shown the expression of eIL-10 (data not shown).

A well-established mouse (CD-1) model of LPS induced PTB was used to evaluate the therapeutic efficacy of eIL-10 (69–72). In order to test the efficacy of eIL-10 in delaying LPS-induced PTB, either phosphate-buffered saline (PBS) or 100 μg of LPS were intraperitoneally injected into pregnant mice on E15. At specific time points (every 2 hours), mice were injected intravenously (tail vein) with either PBS or eIL-10, (500 ng eIL-10 per injection), and PTB was monitored (**Figure 3B**). The dose of 500 ng eIL-10 was chosen based on our preliminary studies and functional data. As shown in **Figures 3C, D**, mice injected with PBS delivered at term (n = 10,125.0 ± 7.0 hours, E-19-20), whereas LPS mice delivered preterm (n = 12, 6 ± 1.5 hours after LPS injection, E-15, P < 0.0001 versus PBS). Co-treatment of eIL-10 in LPS mice delayed preterm labour (n = 8, 20.8 ± 7.8 hours after LPS injection, E-16-17 P = 0.0005 versus PBS, P = 0.072 versus LPS) whereas rIL-10 has shown delayed in preterm labour (n=3, 60 ± 7.8 hours after LPS injection) but didn’t deliver the live pups. Although there is variation in the human parturition which occurs between 37 to 42 weeks of gestation and mouse gestational length from 19-21 days, but delay with eIL-10 is equivalent to almost 10 days in human pregnancy (73).

In another experimental cohort, mice from all treatment groups were sacrificed after 6 hours of treatment to test NF-κB activation in FMs and inflammatory cytokine levels in maternal blood. As shown in **Figures 3E, F**, LPS-induced NF-κB activation was decreased in the FM tissues of mice injected with LPS+eIL-10.

**Figure 4:**
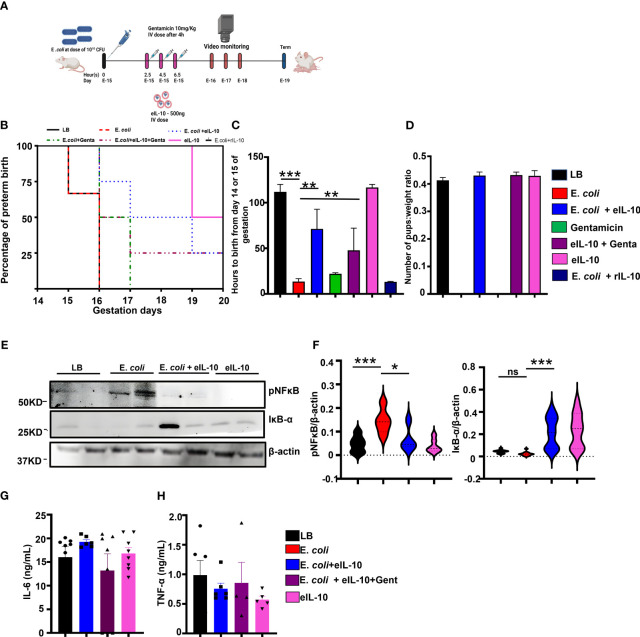
Effect of eIL10 in E. *coli*-induced ascending infection mouse model. **(A)**. Schematic representation of ascending infection-induced preterm birth mouse model and intravenous treatment with eIL10 (500 ng) and/or gentamicin (10mg/kg). Infection is induced by vaginal administration of E. *coli* (10^10^ colony forming units [CFU]). **(B)**. Survival graph comparing liquid broth, LB (black), E. *coli* (red), E. *coli* + eIL-10 (light blue), E. *coli* + gentamicin (green), E. *coli* + eIL-10 + gentamicin (purple), only eIL-10 (pink) and rIL-10 (dark blue) treatment groups. E. *coli* treatment caused PTB within 48 hours, while E. *coli* + eIL-10 delayed PTB to term delivery with live pups whereas gentamicin treatment delays only 24hours compared to E. *coli* group. For all groups, n ≥ 4 **(C)**. Delay in preterm birth (in hours) shown for each treatment group. Color key as seen in **Figure 4B**. A significant delay in preterm birth is shown in mice treated with eIL10 or eIL10 and gentamicin compared to challenge with E*. coli*. **(D)**. Live pups from the eIL-10 and/or gentamicin treatment were weighed and their ratio of the number of pups to their weights were shown in bar graphs. **(E)**. Western blot of fetal membranes collected from pregnant mice 24 hours after treatment. Pregnant mice were treated with vehicle liquid broth (LB), E. *coli*, co-treatment of E*. coli* and eIL10, or eIL10 without E. *coli* challenge. E. *coli*-induced increase of activated NF-κB (phosphorylated: pNF-κB) is reversed with eIL10 administration. IκB-α (an inhibitor of NF-κB is increased with treatment of eIL10. **(F)**. Western blot of fetal membranes showing levels of phosphorylated NF-κB normalized to total NF-κB and IκB-α normalized to β-actin in fetal membranes collected from mice 24 hours after treatment as measured in western blot. Color key as seen in **Figure 4B**. **(G)**. Immune challenge tests: Serum levels of IL-6 production measured by ELISA in pups born after exposure to E. *coli*, eIL10, and gentamicin treatment *in utero*. No significant changes are seen in IL-6 production in pups regardless of exposure indicative of lack of long term immune suppression. **(H)**. Levels of TNF-α production in pups exposed to E. *coli*, eIL10, and gentamicin treatment *in utero* measured by ELISA. Color key as seen in **Figure 4B**. No significant changes are seen in pups’ TNF-α production. For all groups, n ≥ 5. Data are shown as means ± SEM. Data were analyzed using a one-way ANOVA with a Tukey’s post hoc test. *P ≤ 0.05, **P ≤ 0.05 ***P ≤ 0.001. ns represents not significant.

The LPS model of PTB is a massive systemic inflammatory model, and effects both the mother and the fetus. Endotoxemia is not a feature of ascending infection associated human PTB, however, endotoxin accumulation in the amniotic fluid is a major factor associated with fetal inflammatory response and PTB (74)., Our lab has developed a live bacteria-induced ascending infection model of PTB to evaluate the efficacy of eIL-10 in a more clinically and translationally relevant PTB setting (31). In this model, E. *coli* induces PTB in a dose dependent manner, and a low dose of E. *coli* [10^4^ CFU] can produce PTB with live pups like PTB defined in humans (live births before 37 weeks gestation). As depicted in **Figure 4A**, timed pregnant CD-1 mice were challenged with either liquid broth (LB), or E. *coli* (10^10^ CFU) through vaginal administration. After 2.5 hours of E. *coli* challenge, mice were treated with either eIL-10 or Gentamicin alone or in combination with eIL-10 and gentamicin three times at 2-hour intervals. Mice administered with either LB alone or eIL-10 alone delivered at term (n=3, 120 ± 5.0 hrs), whereas E. *coli* challenged mice (on E15) experienced PTB and delivered prematurely (n=10,13.50± 3.2 hrs) (**Figures 4B, C**). Interestingly, mice treated with eIL-10 (n=8, 71.25 ± 11.58) or in combination with gentamicin (n=6, 47.75 ± 14.30 hrs) showed substantial delay in PTB induced by ascending infection. Surprisingly, treatment with gentamicin alone has not shown any substantial delay in PTB (n=8,10.5 hrs), which is consistent with human clinical studies with antibiotics. Newborn pups from the different treatment groups were weighed and as shown in **Figure 4D** there is no change in the pups weight among different groups. In summary, eIL-10 administration delayed PTB in two models of infection during pregnancy. In a clinically relevant ascending model of PTB, live pups were delivered after eIL-10 alone or eIL-10 gentamicin, suggesting that eIL-10 may be beneficial as an adjunct therapeutic agent, along with antibiotics.

In order to determine the *in utero* mechanistic action of eIL-10, another cohort of mice were sacrificed after 24 hours of LB and E. *coli* and eIL-10 co-treatment to collect FM. As shown in **Figures 4E, F**, E. *coli*-induced NF-κB activation was significantly decreased with eIL-10, and IκB degradation was inhibited by eIL-10 treatment. These results suggest that eIL-10 has improved the availability of IL-10 targeted sites, and demonstrated anti-inflammatory activity by inhibiting NF-κB activation.

Long term immunosuppression is one of the major concerns with an anti-inflammatory therapy during pregnancy and any other condition, especially when they functionally affect transcription factor NF-κB. Neonatal immunosuppression, if any, after eIL-10 treatment was further tested. The live pups delivered at term by LB, eIL-10 treatment or gentamicin were challenged with LPS to determine the effect of fetal exposure to eIL-10, or drug treatments on fetal immune response. As shown in **Figures 4G, H**, there were no differences in the production of pro-inflammatory cytokines IL-6 and TNF-α, suggesting that *in utero* eIL-10 exposure does not lead to immune suppression during the neonatal period.

### 3.4 eIL-10 inhibits NF-κB -mediated signaling

**Figure 5:**
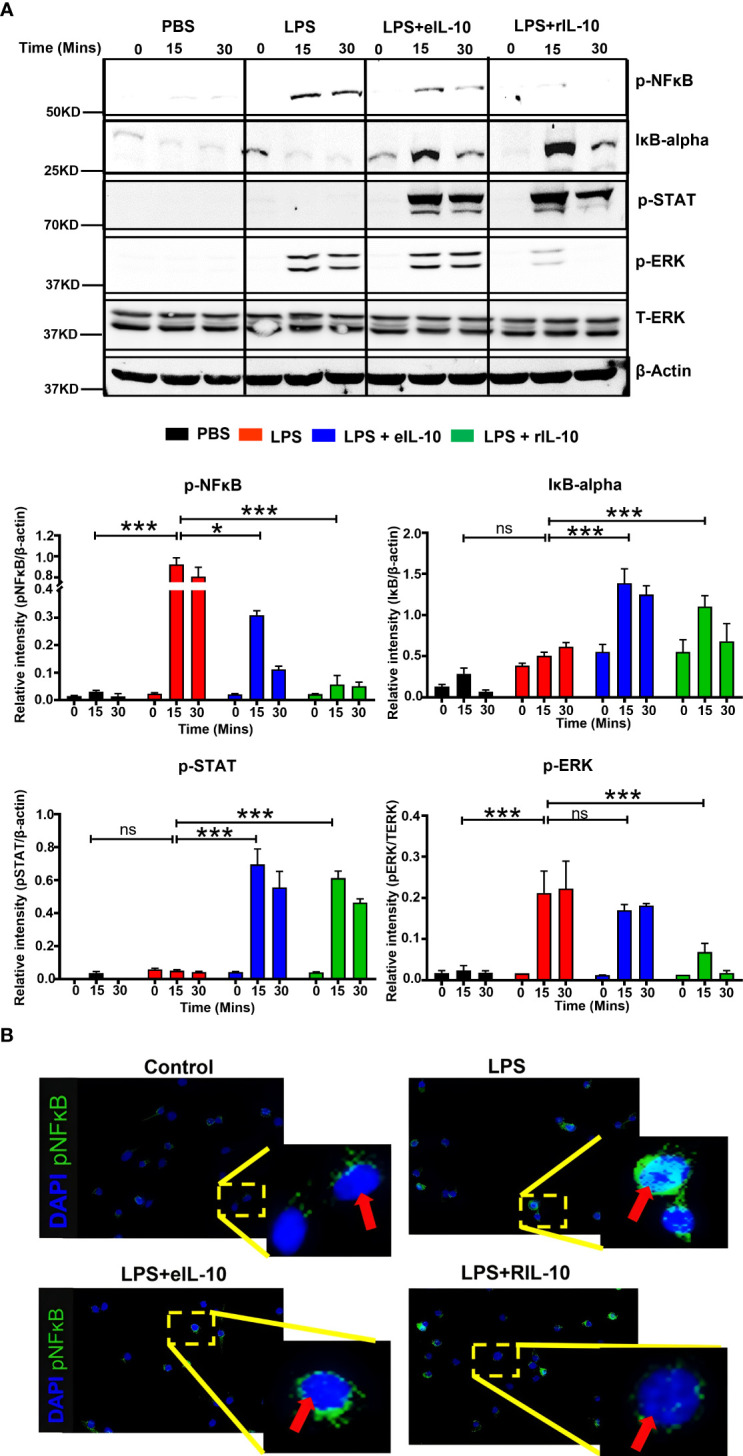
eIL-10 inhibits NF-κB pathway. **(A)**. Representative western blot images of cell lysates collected from RAW264.7 (mouse macrophage) cells treated with LPS (red bar) and co-treated with either eIL10 (blue bar) or rIL10 (green bar) for a treatment time of 0, 15, or 30 minutes. LPS-induced increases of activated NF-κB (phosphorylated: p-NF-κB) and activated ERK (phosphorylated: p-ERK) were reversed by treatment with eIL10 or rIL10. Co-treatment of eIL10 or rIL10 also increased levels of IκB-α (inhibitor of NF-κB) and phosphorylated STAT3 (p-STAT3). Bar graphs show relative intensity of these bands compared to β-actin, and phosphorylated ERK shows relative intensity against total ERK. All the experiments were repeated three times. Normalized data were shown as means ± SEM. Data were analyzed using a one-way ANOVA with a Tukey’s post hoc test. *P ≤ 0.05, ***P ≤ 0.001, ns represents not-significant. **(B)**. Immunocytochemistry staining of RAW264.7 cells with activated NF-κB (phosphorylated: pNF-κB) antibody (green) and DAPI (blue) to show translocation of p-NF-κB into the nucleus.

Although we showed in **Figures 3** and **4** that eIL-10 delivery inhibited *in vitro* and *in vivo* NF-κB activation, we next evaluated whether eIL-10 administration blocked inflammation-associated signaling pathways. After determining the pharmacological effect of eIL-10 and the initial mechanism in tissue samples, we further investigated the detailed molecular mechanism of eIL-10 in mouse macrophage cell lines RAW264.7. Cells were treated with LPS (100 ng/mL) and either eIL10 or rIL10 (500 ng/mL each) for 15 or 30 minutes, and cell lysates were harvested. rIL-10 was used in these experiments to confirm the mechanistic activities and to compare the functions of rIL-10 in EVs vs free IL-10. Lysates were probed for signaling molecules in the NF-κB activation pathway by western blotting. As shown in **Figure 5A**, eIL10 and rIL10 both show a reduction of LPS-activated NF-κB and increased expression of IκBα, an NF-κB inhibitor, and phosphorylated STAT3, which is an anti-inflammatory molecule that works by inhibiting the NF-κB pathway (75).

The effect of IL-10 on the nuclear translocation of activated NF-κB was also determined in RAW264.7 cells. As a transcription factor, NF-κB exerts its inflammatory effects in the nucleus after binding to the kB motif of proinflammatory gene promoters. Phosphorylated NF-κB and cell nuclei were immunostained after treatment with either LPS, LPS+eIL-10, or rIL-10. As shown in **Figure 5B**, LPS increased the nuclear translocation of phosphorylated NF-κB; however, co-treatment with eIL10 or rIL10 blocked the entrance of NF-κB. These data suggest that, regardless of the mechanistic signals generated, both eIL-10 and rIL-10 block NF-κB activation and prevent its nuclear translocation.

### 3.5 eIL-10 treatment reduced neutrophil infiltration and histologic chorioamnionitis

**Figure 6:**
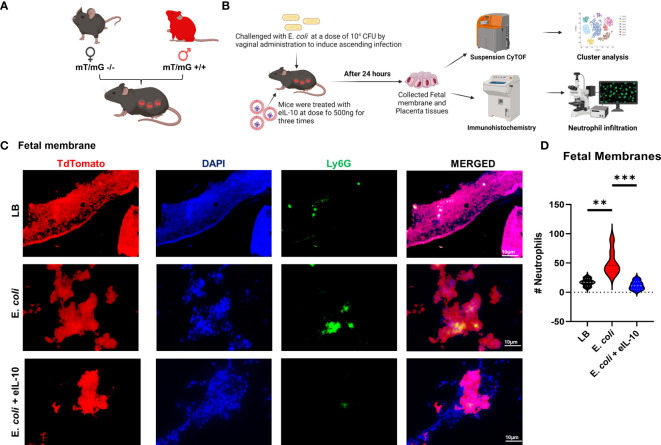
eIL10 treatment reduced neutrophil infiltration and HCA. **(A)**. Schematic representation of a transgenic animal model with a membrane-targeted, 2-color fluorescent Cre-reporter allele where membrane-expressed tandem dimer Tomato (tdTomato—mT+). mT+ males were mated with wild-type female (WT) mice, all fetal tissues had the mT/mG construct expressing mT+ (red fluorescence), keeping maternal tissues negative. **(B)**. Schematic representation of E. *coli* challenge and treatment of mice, various approaches of analysis of samples and data collection. Using the pregnant mouse model **(A)**, mice are treated with vaginal administration of 10^4^ colony forming units (CFU) of E. *coli* to induce ascending infection. Co-treatment with eIL10 is administered at a dose of 500 ng (intravenous through tail vein). **(C)**. Immunohistochemistry of fetal membranes 24 hours after administration of vehicle liquid broth (LB), E. *coli* challenge, co-treatment of E. *coli* with eIL10, or eIL10 without E. *coli* challenge. Tissues were stained with Ly-6G (green) for neutrophils and DAPI (blue) to show nuclei. TdTomato (Red) was expressed by the tissues. **(D)**. Violin graphs show significant reduction in neutrophil levels by eIL10 (blue) after challenge with E. *coli* (red) as seen in IHC fluorescent microscopy. The number of neutrophils in fetal membranes is reduced to levels similar to those seen in vehicle liquid broth (LB, black). For all groups, n ≥ 5. Data are shown as means ± SEM. Data were analyzed using a one-way ANOVA with a Tukey’s *post hoc* test. **P ≤ 0.01, ***P ≤ 0.001.

Neutrophil infiltration into the amnio chorionic membranes is the hallmark of histologic chorioamnionitis (HCA) and fetal inflammatory response. The role of fetal neutrophils is well established and reported (31, 76). HCA is a major determinant of pregnancy outcomes, as well as an indicator of neonatal morbidities (77, 78). To further elucidate the mechanism of eIL-10 in reducing fetal inflammatory response and HCA, we used a transgenic mouse model with a membrane-targeted, 2-colour fluorescent Cre-reporter allele (mT/mG construct), where mT (red fluorescence) is expressed in all cells (57). Female wild-type mice were mated with homozygous males (C57BL/6J) with mT construct, resulting in progeny with mT expression seen exclusively in fetal, but not maternal, cells (**Figure 6A**). These mice were then challenged with E. *coli* and treated with three doses of eIL10 (500 ng). FMs were collected after 24 hours (**Figure 6B**). Neutrophils were stained with lymphocyte antigen 6 complex locus G6D (Ly6G) antibody in the mT-expressing FMs from LB, E*. coli* and E*. coli* + eIL-10 (**Figure 6C**). E. *coli* treated animals had a significant increase in the number of Ly6G+ cells (neutrophil marker) in the FMs (**Figure 6D**), compared to LB (p=0.004). Co-treatment with eIL-10 significantly reduced neutrophil infiltration compared to E. *coli* alone (P=0.0003). A similar reduction in immune cell infiltration was also seen in the placental tissues (**Supplementary Figure 3**).

Based on these data, we report that eIL-10 mediated reduction in immune cell infiltration is associated with delayed ascending infection-induced PTB in the mouse models used in this study.

### 3.6 eIL-10 treatment reduced fetal inflammatory response

PTB is characterized by the premature activation of pro-inflammatory response at the feto-maternal interface (79, 80). It is well established that fetal inflammatory response overrides feto-maternal immune tolerance, leading to PTB (81, 82). In order to determine the effect of eIL-10 on fetal inflammatory response, mT+ mated C57B6/J mice were treated with either LB, E. *coli* (10^4^ CFU) or E. *coli* +eIL-10. FMs were collected after 24 and 48 hours. The model of ascending infection in the C57B6/J is shown in **Supplementary Figure 4A** and dose determination is shown in **Supplementary Figure 4B**. Single cell suspension from collected FMs was subjected to mass cytometer (Cytometer Time of Flight (CyTOF)) to comprehensively phenotype infiltrated immune cells. We therefore stained the single-cell suspension of mouse FMs with a panel of 25 metal-tagged antibodies against cell surface markers and intracellular markers (**Supplementary Table 2**).

**Figure 7:**
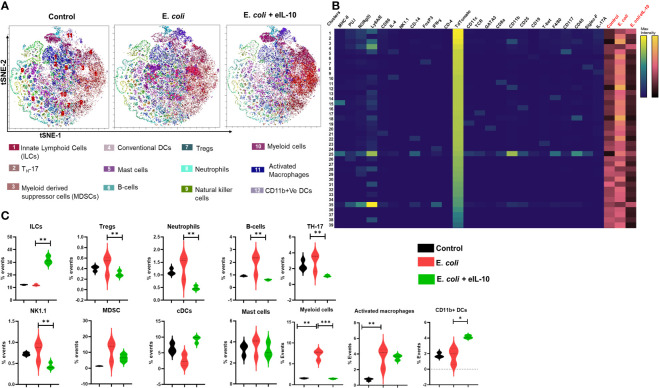
Fetal specific Immune cell characterization during pregnancy in different treatment groups after 24 hours by mass cytometer. **(A)**. Identification of differentially distributed cellular phenotypes by t-SNE in concatenated control, E. *coli* and E. *coli* + eIL-10 treated groups, respectively. **(B)**. The heatmaps shows the relative intensity of each parameter for different clusters in different groups. **(C)**. The percentage events of the particular cluster were determined by the bar charts from the cluster explorer tool and cluster was characterized by the expression profile expression markers. The characterized clusters percentage events were compared between different treatment groups. For all groups, n ≥ 5. Data are shown as means ± SEM. Data were analyzed using a one-way ANOVA with a Tukey’s *post hoc* test. *P ≤ 0.05, **P ≤ 0.01, ***P ≤ 0.001.

**Figure 8:**
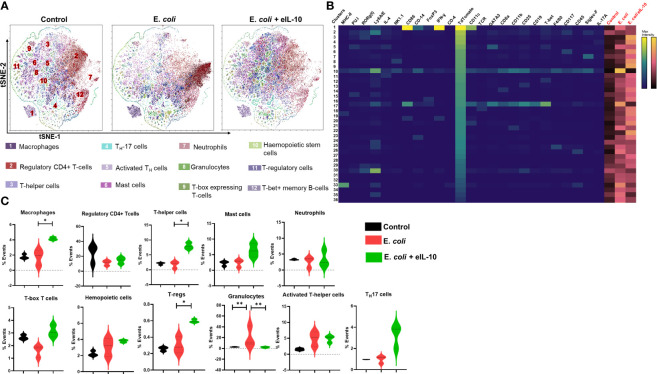
Fetal specific Immune cell characterization during pregnancy in different treatment groups after 48 hours by mass cytometer. **(A)**. Identification of differentially distributed cellular phenotypes by t-SNE in concatenated control, E. *coli* and E. *coli* + eIL-10 treated groups, respectively. **(B)**. The heatmaps shows the relative intensity of each parameter for different clusters in different groups. **(C)**. The percentage events of the particular cluster were determined by the bar charts from the cluster explorer tool and cluster was characterized by the expression profile expression markers. The characterized clusters percentage events were compared between different treatment groups. For all groups, n ≥ 5. Data are shown as means ± SEM. Data were analyzed using a one-way ANOVA with a Tukey’s *post hoc* test. *P ≤ 0.05, **P ≤ 0.01.

We used t-Stochastic Neighbour Embedding (t-SNE) to visualize high-dimensional CyTOF data on a two-dimensional scatterplot, where cells with a similar expression of surface markers are grouped together (83). We applied a K-nearest-neighbour density-based clustering algorithm called Phenograph to explore the phenotypic diversity of immune cell populations in the different groups of mice FM tissues (84). This algorithm allows the unsupervised clustering analysis of data from single cells after 24 and 48 hours, as represented in **Figures 7A** and **8A** respectively. The unsupervised clustering of single cells from the FM tissues of control, E*. coli* and E*. coli*+eIL-10 samples created a detailed tSNE map of distinct cell populations with individual immune cell markers (**Supplementary Figure 5**). The output was organized using a cluster explorer tool to visualize the phenotypic continuum of cell populations. The heat maps generated by the cluster explorer show the relative intensity of each parameter for a given cluster in different treatment groups for 24 and 48 hours and are depicted in **Figures 7B** and **8B**. The fetal specific immune response detected as TdTomato expressing cells (assessed by anti-TdTomato antibody conjugated to 175Lu) showed a relatively higher incidence of immune cell markers in the FMs of mice with E. *coli* infection after 24 hours compared to the group treated with eIL-10. This analysis revealed distinct CD11b+ myeloid populations in the E. *coli* and E. *coli*+eIL-10 treated FMs that were absent in the control group. The cluster size, as a percentage of events, across the different treatment groups, and at both 24- and 48-hours post-treatment, are shown in violin graphs: **Figures 7C** and **8C**. After 24 hours post-treatment, several pro-inflammatory immune cell populations were significantly more abundant (p-value < 0.05) in the infection group, and significantly less abundant in the eIL-10 treated group compared to controls. Specifically, Foxp3 + cells (Tregs), CD19+ (B cells), NK1.1+ (NK cells) and Ly6G+SiglecF+ (neutrophils).

**Figure 9:**
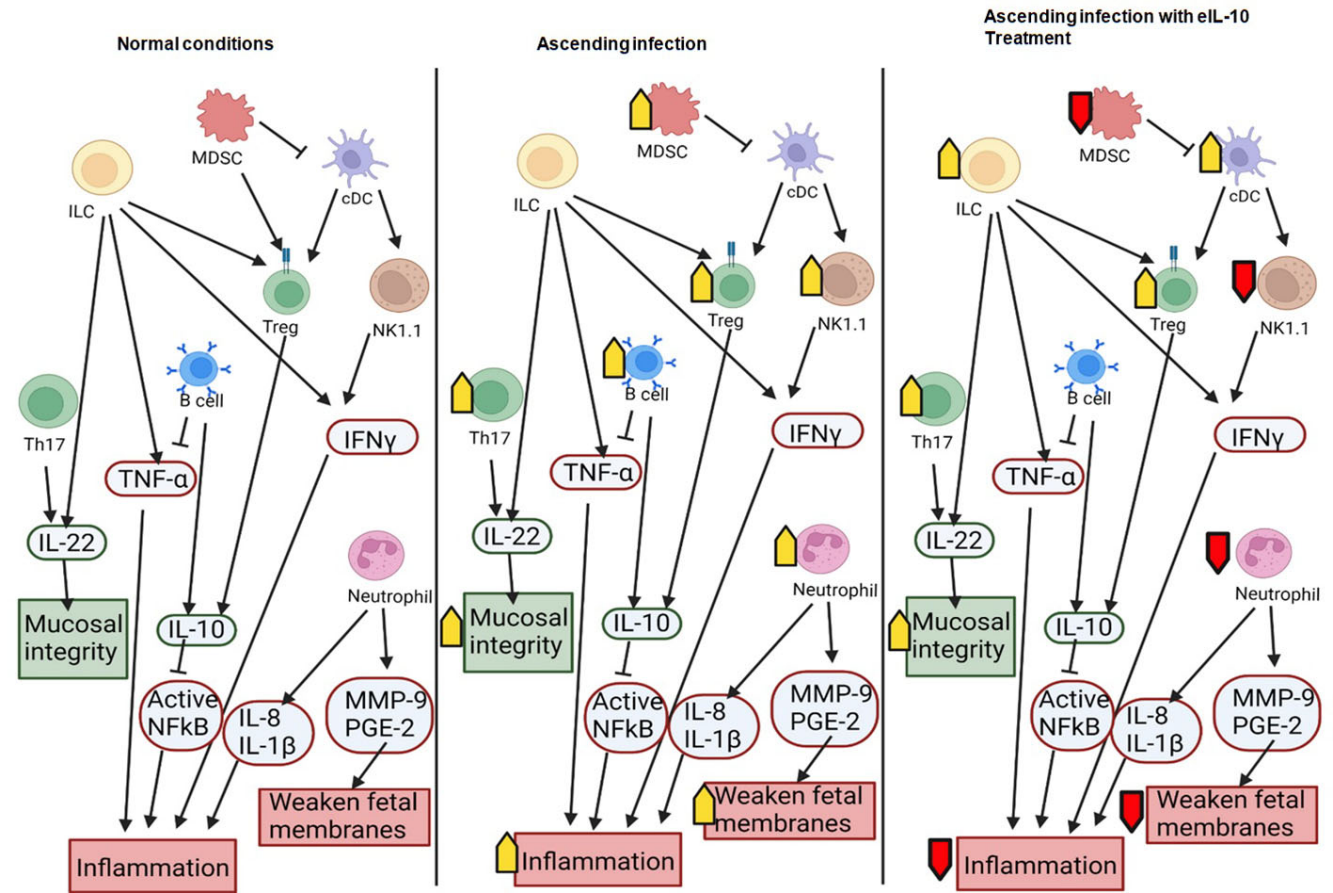
Schematic representation of the interactions between immune cells and their roles in pregnancy and ascending infection. Anti-inflammatory molecules and effects that maintain pregnancy are shown in green and pro-inflammatory molecules or effects that can lead to preterm birth are shown in red. Increased activity in a condition (such as ascending infection or treatment with eIL10) is marked with a yellow arrow, and decreased activity is marked with a red arrow.

Interestingly, eIL-10 treatment produced a higher frequency of CD45, T-bet, CD25, CD117 and RORγ(t) cells. Although further characterization would be needed to confirm classification, this expression pattern is consistent with Innate lymphocyte type of cells (ILC-like). ILCs are innate lymphocytes that play important roles in immune defence against microbes, the regulation of adaptive immunity, tissue remodelling, and the repair and homeostasis of haematopoietic and nonhaematopoietic cell types (85–88). ILCs were also reported as one of the key sources of IL-10. Bando et al. have shown that IL-10 secreted by ILCs might activate a feedback loop that amplifies production (89). As illustrated in the **Figure 9** eIL-10 treatment thus increases ILCs, which may in return produce IL-10 to provide a positive feedback mechanism to involve in the tissues repairing and antimicrobial action in E. *coli* induced PTB. We analysed placenta using CyTOF and determined that the infiltration of immune cells is not as dominant as in FMs, and that eIL-10 minimally impact this infiltration. This is not unexpected, as infection associated PTB has a minimal effect in causing placentitis. These data show that the co-treatment of eIL0 reduces pro-inflammatory immune cell influx into the FM. 

## 4 Discussion

In this study, we provide both proof of the concept, and pharmacological evidence for a protective role of IL-10 ascending infection-mediated PTB by leveraging a novel mode of exosomal delivery of the anti-inflammatory cytokine. rIL-10 could be successfully incorporated into EVs (eIL-10) using electroporation with a loading efficiency of ~ 70%. Importantly, electroporation did not alter exosomal size, structure, or tetraspanin marker expression while maintaining the functional properties of encapsulated IL-10. Moreover, eIL-10 could cross placental, and FM feto-maternal interface barriers compared to rIL-10, which was most likely degraded or lost due to a short half-life. eIL-10 was taken up by both maternal and fetal cells. Mechanistically, eIL-10 signalled through the STAT3-SOCS3 pathway to inhibit NF-κB activation, which subsequently reduced inflammatory cytokine release from cells. PTB induced by an ascending infection is usually associated with fetal inflammatory response and HCA. Both eIL-10 and a combination of eIL-10 and gentamicin delayed PTB and resulted in term birth with live pups. Although, combination of eIL-10 and gentamicin delayed PTB and results in the live pups, further optimization of the doses and frequencies are required since the combination therapy has shown non-significant lower activity than eIL-10 alone. The protection against PTB was associated with a reduction in fetal inflammatory response and HCA. We suggested that eIL-10 is a safe mode of treatment against PTB, because it did not cause long term immunosuppression either in in the neonate, as indicated by their active immune response to a challenge by an infectious stimulus.

Our primary goal was to plan a strategy with the ‘fetus as a patient’ as the focus to reduce fetal inflammatory response contributing to PTB. To test this, we recreated an ascending infection model to test the efficacy of eIL-10 as seen in human PTB. Maternal systemic infection and endotoxaemia are rare in human PTB, suggesting that infection is primarily restricted to an ascending tract from the vagina to amniotic cavity (5, 90). Bacterial colonization and infection elicit an inflammatory reaction such as HCA, and increased levels of neutrophil infiltration, IL-6 and TNF-α, which are some of the classic signs of amniotic infection and inflammation (13, 91). IL-6 facilitates the production of sustained and sufficient inflammation during PTB and innates immune responses by mediated by neutrophils, macrophages, and lymphocytes (92). TNF-α is considered as one of the early mediators of the inflamed tissue and involved in the regulation of macrophages (93). Our findings support the clinical classification of fetal inflammatory response based on the presence of infiltrating neutrophils, while also providing a rationale for our inclusive approach to encompass a broader spectrum of acute inflammatory lesions in the placental and fetal membranes. Besides, eIL-10 reduces maternal inflammation. By controlling both feto-maternal inflammation, we postulate that eIL-10 can effectively reduce the incidences of infection associated preterm birth.

This study showed that eIL-10 does not demonstrate any postpartum immunosuppressive effects in the offspring. The immunosuppressive effects observed were transient and sufficient to delay infection and inflammation associated PTB, however, they were restored after delivery. This could be due to the paracrine targeting abilities of EVs. The adhesion molecules found on some mesenchymal stem cell derived EVs allow them to directly target sites of inflammation (94). Their accumulation in inflamed tissues has been cited in acute kidney injury and intracerebral hemorrhage (95, 96). It has also been shown that EVs derived from dendritic cells specifically target activated T cells, but not resting T cells (97). It is not currently known whether HEK-derived EVs, which were used in this study, have similar targeting properties. Based on our results, we propose that they do elicit paracrine targeting ability. If acute inflammation is present in pregnant mouse, eIL10 may target that tissue and inhibit the inflammation. If inflammation is not present, there may be concerns that eIL10 will potentially generate toxic effects by releasing IL-10 in tissues that are not inflamed. Our mT+ model allowed us to determine the effect of eIL-10 in reducing HCA where fetal specific inflammatory cells were localized on FMs and placenta. Suspension CyTOF analysis indicates that eIL-10 inhibited the influx of fetal immune cells into the fetal membranes within 24 hours; however, membranes regained the inflammatory capacity within 48 hours. This inflammatory membrane activation is essential for propelling the process required to generate maternal inflammatory amplification and to tilt the homeostatic balance to enable labour (98). Although we focused specifically on the reduction of fetal inflammatory response, maternal immune cells also contribute to HCA (82, 99, 100); however, the overall reduction of the inflammatory activation suggests that both fetal and maternal inflammatory processes are reduced by eIL-10 treatment.

Exosomal encapsulation of IL-10 has been reported by Tang et al (101), who engineered macrophages to successfully treat ischemic acute kidney injury. Among the different loading techniques like (Incubation, Sonication and electroporation), we used electroporation to incorporate IL-10 in human derived EVs to improve the loading efficiency. All *in vivo* model studies were conducted after confirming loading efficiency, lack of cytotoxicity, and ability to inhibit NF-κB activation and inhibition of cytokine release. In this study, we used rIL-10 for comparison purposes while testing eIL-10. rIL-10 is an effective anti-inflammatory agent, and its effectiveness in various reproductive tissues has already been shown (102, 103). We first examined the mechanisms of action and differences, if any, between the rIL-10 and its encapsulated version (eIL-10) (**Figures 5A, B**). Activation of TLR4 in response to a Gram-negative bacterial stimulus recruits adaptor proteins such as MyD88, and MyD88 adapter-like protein (Mal) (104). Mal then interacts with the tumour necrosis factor receptor-associated factor (TRAF) (105), which leads to phosphorylation of Akt (106), activation of IKK (107), and activation of MAPK (108). Activation of IKK leads to phosphorylation of IκB and dissociation of the IκB-NFκB complex (109). This allows NF-κB to translocate to the nucleus, where it can alter transcription of specific genes (110). TRAF6 also activates ERK MAPK pathways (111), which further activates IKK and increases NF-κB activation (112). Extracellular IL-10 can inhibit this inflammatory pathway when it interacts with the membrane receptor IL-10R. Activation of this receptor results in the phosphorylation of STAT3 (75), which can enter the nucleus and bind to the SOCS3 promoter, inducing transcription of SOCS3 (113). SOCS3 reduces inflammation in multiple ways. First, it induces the degradation of Mal on TLR4 to block the LPS-induced inflammation (114) and causes degradation of TRAF6 (115). It also prevents the activation of ERK (116) and degrades NF-κB p65 (117) (**Figure 5C**). All these targets help to reduce inflammation within the cell, and this pathway has been confirmed by data in this study.

One of the objectives of this study was to identify differences in efficacy and mechanism of action between eIL10 and rIL10. EVs and their cargo can facilitate its function in multiple ways: (1) EVs can enter the recipient cell by endocytosis, (2) EVs can release their cargo outside the cell environment, and (3) EVs can enter the cell through an exosomal surface receptor and release their cargo inside. Our data suggest that eIL-10 may be exerting its action in multiple ways based on the western blot analysis, as shown in **Figure 5A**. It is expected that eIL-10 could enter the cell by endocytosis and act intracellularly, rather than acting *via* the extracellular IL-10 receptor. However, the activation of STAT3 by eIL-10 (like that seen for rIL-10), which is recruited to IL-10R for phosphorylation, suggests that IL-10 may also be released extracellularly, so that IL-10 can bind to IL-10R (75). According to our data, the main difference between the two treatments is that eIL10 showed phosphorylated (p) ERK, whereas p-ERK expression was not observed after rIL-10 treatment. eIL-10 treatment also did not activate the MAPK pathway like rIL-10, as shown in **Supplementary Figure 1C**. The differences observed could very well be explained by the differential activation of signaling pathways by packaged IL-10 and free IL-10. rIL-10 action is restricted to receptor-mediated signaling, whereas eIL-10 could cause receptor-mediated signaling as well as signaling inside the cytoplasm after entering the cells and the release of its cargo in the cytoplasm. IL-10 in the cytoplasm of the recipient cell is expected to generate mechanistic events that can involve signaling molecules independent of ligand-receptor signaling. EVs deliver cargo to the recipient cell in different ways (ligand-receptor mediated entry of EVs into the cell, endocytosis of EVs and delivering cargo in the cytoplasm, delivering cargo outside and taken up by a cell’s cognate receptors, etc.). We speculate that eIL-10 may function in multiple ways in a recipient cell, initiating distinct pathways. Alternatively, the rIL-10 effect may be limited due to its short half-life, and the activation of several molecules are not observable after 15 minutes. It is likely that sustained stimulation may be required to see changes in the signaling marker expressions.

It is known that packaging drugs inside EVs protects them from degradation and extends their half-life, but if eIL10 is releasing its cargo outside the cell, it should be acting and degrading at the same rate as rIL10. It is possible that EVs are acting like a hydrogel to allow gradual release of the cargo, either outside or inside. A hydrogel is a three-dimensional network of polymers that absorbs biological fluids to control and sustain drug release at a target site. They have been developed using nanoparticles and liposomes, but not EVs. These hydrogels provide the benefit of a biodegradable scaffolding material gradually releasing a drug over a period of days or weeks, and then degrading after use. This form of drug delivery is especially useful in cytokine therapy because it overcomes the limitation of their short half-life, while also preventing systemic toxic effects (118). This would explain the differences in treatment time between the two molecules. The sustained effect seen beyond that expected with rIL-10 suggests that EV delivery of natural biologicals may be better than nanoparticle- and liposome-mediated approaches.

Another difference between eIL10 and rIL10 treatment was the effect on ERK. Cells treated with rIL10 showed a significant reduction in levels of p-ERK, however, treatment with eIL10 did not reduce p-ERK. As the rest of the molecules in these pathways show very similar responses to the different treatments, it is unclear why a specific difference with p-ERK would be seen. It may be that eIL10 activates an additional pathway in the cell that could interact with the ERK pathway, but this would require further experiments to confirm. It is also possible that the difference is due to timing. When looking at the effects of rIL10 on other molecules, rIL10 shows the complete inhibition of NF-κB and it reaches maximum levels of p-STAT3 and p-IκB in 15 minutes. By 30 minutes, these effects showed no change or were even beginning to reverse. p-ERK is more gradually reduced with rIL10 treatment, however, suggesting that this is a slow progressing pathway. As rIL10 is acting at a faster rate than eIL10, it is possible that eIL10 would show a similar reduction of p-ERK if measured over a longer time period.

We were able to overcome some of the limitations of our prior studies using the exosomal delivery of an NF-κB inhibitor. In addition to its independent effect in delaying PTB, eIL-10 can be an excellent adjuvant drug along with antibiotics to reduce infection and inflammation, and to prolong pregnancies to term. This is likely to be the scenario in human PTB as the elimination of an infectious agent is required to avoid any overgrowth or systemic spread of microorganisms. In summary, we report the maternal administration of an exosomal drug that can minimize fetal inflammatory response and HCA, delay PTB, and in most instances lead to term delivery.

EVs as a drug delivery vehicle overcome a major limitation in drug development and testing during pregnancy where placenta and FMs act as drug barriers to address fetal responses. Our approach supports the hypothesis that addressing the terminal events where the mother is considered as a patient, without addressing the fetus as a patient, may not be sufficient to reduce PTB risk. Although this study has potential therapeutic implications to treat preterm birth it has its own limitations. 1) Animal experiments were conducted in the small samples size with n=8-10. 2) Using single dose of the gentamicin in combination of eIL+10 treatment and lack of cell phenotype and histology data from the eIL-10 + gentamicin mice. In the current study we used gentamicin due to its documented effectiveness against gram-negative bacteria including E. *coli* commonly associated with ascending infections. Further investigations need to be carried to explore the alternative antibiotic regimens, including ampicillin, amoxicillin + clavulanic acid and ampicillin + sulbactam. 3) The lack of evaluation or comparison with cervicovaginal inflammatory milieu, 4) Utilization of spin filters for EV purification may not be as effective as other techniques that are recently developed and proposed in the literature (119–122). Including additional purification that can include gradient based precipitation of EVs and QC measures will be required for consistency when large scale productions are needed for clinical trials. Regardless, as shown in **Figure 1D**, we were still able to get EVs of expected size and able to have high efficiency cargo loading (~73%) in our EVs. We were also able to generate valuable information regarding the association of rIL-10 with EVs. This highlights the need for caution when interpreting our findings, particularly in terms of cargo loading into EVs, as spin filters might not capture the full spectrum of EV populations. Further investigations employing more advanced purification methods are warranted to validate and expand upon our initial observations. In summary, we would like to emphasize that the numerous challenges associated with delivering recombinant IL-10 to fetal compartments to mitigate the fetal inflammatory response can be overcome by encapsulating it within a vehicle capable of crossing these barriers, such as extracellular vesicles. This approach not only enhances its stability and bio-availability but also prolongs its lifespan within the targeted compartments.

### 4.1 Statistical analysis

Statistical analysis was performed using the GraphPad Prism 9.7 software (GraphPad, San Diego, CA). The statistical parameters associated with each figure are reported in the figure legends. All data are reported as the mean ± SEM. Statistical significance in differences between experimental groups was assessed as follows: unpaired t-test for ELISA and ExoView, Fisher’s exact test for the rates of preterm birth and pup mortality, and Mann–Whitney U-test for gestational age. All other statistical comparisons were carried out using two-way ANOVA. The following notations were used in all figures: *P < 0.05, **P < 0.01, and ***P < 0.001. Significance was considered at P < 0.05.’

## Data availability statement

The original contributions presented in the study are included in the article/**Supplementary Material**. Further inquiries can be directed to the corresponding author.

## Ethics statement

The animal study was reviewed and approved by Institutional Animal Care and Use Committee (IACUC) at the University of Texas Medical Branch, Galveston with approved protocol number 041107F.

## Author contributions

AK: Data curation, visualization, methodology, writing- original draft preparation. AM: Data curation. ERad: Methodology. ERow: Data curation, analysis. NV: Data curation. SF: Supervision. SS: Reviewing and editing. MS: Analysis and visualization. RM: Conceptualization, methodology, writing- reviewing and editing. All authors contributed to the article and approved the submitted version.

## Supplementary material

Supplementary Figure 1
**(A)**. Representative western blot images showing the presence of ESCRT protein TSG101 in eIL10 and Electroporated EVS without IL-10. **(B)**. Similarly, the presence of IL-10 was verified using western blotting. **(C)**. Signaling molecules of MAPK kinase pathway and STAT3 pathway in RAW264.7 cells after treatment with LPS at 500ng/mL, eIL-10 and rIL10 at 100 or 500ng/mL, as shown by western blot. Phosphorylated forms of NF-κB, AKT, IκB-α and ERK are shown to increase with *E. coli* challenge. Phosphorylated STAT3 (pSTAT3) increases with treatment of eIL10 or rIL10. Total ERK (TERK) is used to normalize phosphorylated ERK (pERK) levels. β-actin is used to normalize the other molecules.

Supplementary Figure 2
**(A)**. Differentially dyed EVs. A. Schematic representation of the production of red and green stained eIL10, consisting of FITC-conjugated IL-10 loaded into DiD stain EVs. **(B)**. Schematic representation of live imaging of eIL10 distribution in pregnant mice at day E15 compared to distribution of IL-10 that is not loaded in EVs, using stained EVs and eIL10 described in **Supplemental Figure 1A**.

Supplementary Figure 3
**(A)**. Immunohistochemistry of placenta 24 hours after different treatments (Control, E. *coli*, E. *coli*+eIL-10 and eIL-10). Tissues were stained for Ly6G (green) to show neutrophils and DAPI (blue) to show nuclei. **(B)**. Violin graphs show significant increase in neutrophil levels after challenge with E. *coli* as seen in IHC fluorescent microscopy. (Control liquid broth in black, E. *coli* in red, E. *coli*+eIL-10 in blue and eIL-10 alone in pink) For all groups, n ≥ 5. Data are shown as means ± SEM. Data were analyzed using a one-way ANOVA with a Tukey’s *post hoc* test. *P ≤ 0.05, **P ≤ 0.01, ***P ≤ 0.001, ****P ≤ 0.0001.

Supplementary Figure 4
**(A)**. To determine dose of E. *coli* in ascending infection-induced PTB, on gestation day 15, pregnant C57BL/6J mice were challenged with different doses of *E. coli* though vaginal administration. PTB was monitored under video recording. **(B)**. Compared to LB (liquid broth) control animals, vaginal administration of 10^9,^ 10^7^ and 10^5^ CFU E. *coli* resulted in PTB within 36 hours, but delivered dead pups. However, 10^4^ CFU dose induced PTB within 48 hours and delivered PTB live pups which is clinically relevant model to humans. The 10^3^ CFU group showed 50% PTB (2/4). For all groups, n ≥ 10. Data are shown as means ± SEM. Data were analyzed using a one-way ANOVA with a Tukey’s *post hoc* test. *P ≤ 0.05, **P ≤ 0.01, ***P ≤ 0.001, ****P ≤ 0.0001.

Supplementary Figure 5 Representative phenographs of individual markers among different groups of the FM tissues obtained by FlowJo Phenograph plug ins CyTOF data analysis.
