# Supplementary figures and images for: Extracellular Vesicles-mediated recombinant IL-10 protects against ascending infection-associated preterm birth by reducing fetal inflammatory response
Source: Front Immunol. 2023 Aug 4;14:1196453. doi: 10.3389/fimmu.2023.1196453 (PMC10437065; doi:10.3389/fimmu.2023.1196453)

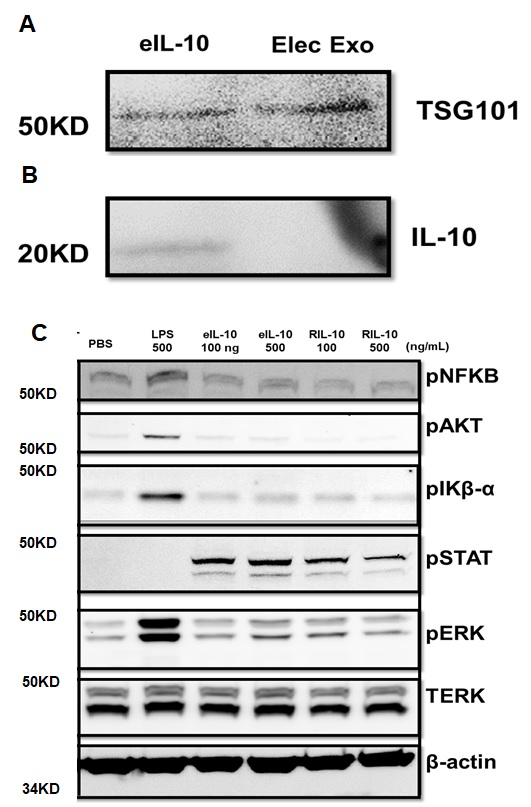

Supplement: Supplementary Figure 1 — (A). Representative western blot images showing the presence of ESCRT protein TSG101 in eIL10 and Electroporated EVS without IL-10. (B). Similarly, the presence of IL-10 was verified using western blotting. (C). Signaling molecules of MAPK kinase pathway and STAT3 pathway in RAW264.7 cells after treatment with LPS at 500ng/mL, eIL-10 and rIL10 at 100 or 500ng/mL, as shown by western blot. Phosphorylated forms of NF-κB, AKT, IκB-α and ERK are shown to increase with E. coli challenge. Phosphorylated STAT3 (pSTAT3) increases with treatment of eIL10 or rIL10. Total ERK (TERK) is used to normalize phosphorylated ERK (pERK) levels. β-actin is used to normalize the other molecules. [file Image_1.jpeg]

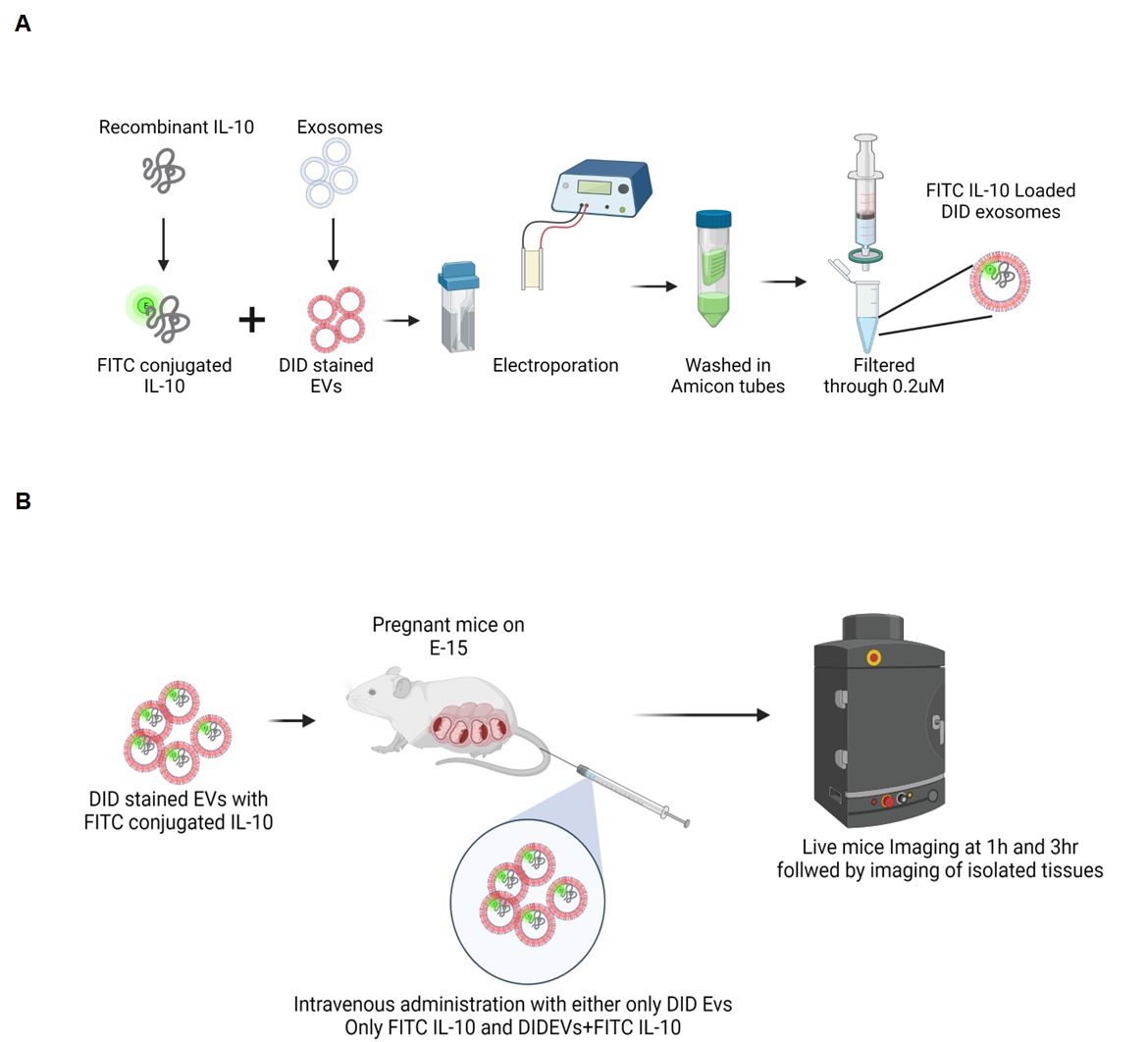

Supplement: Supplementary Figure 2 — (A). Differentially dyed EVs. A. Schematic representation of the production of red and green stained eIL10, consisting of FITC-conjugated IL-10 loaded into DiD stain EVs. (B). Schematic representation of live imaging of eIL10 distribution in pregnant mice at day E15 compared to distribution of IL-10 that is not loaded in EVs, using stained EVs and eIL10 described in Supplemental Figure 1A . [file Image_2.jpeg]

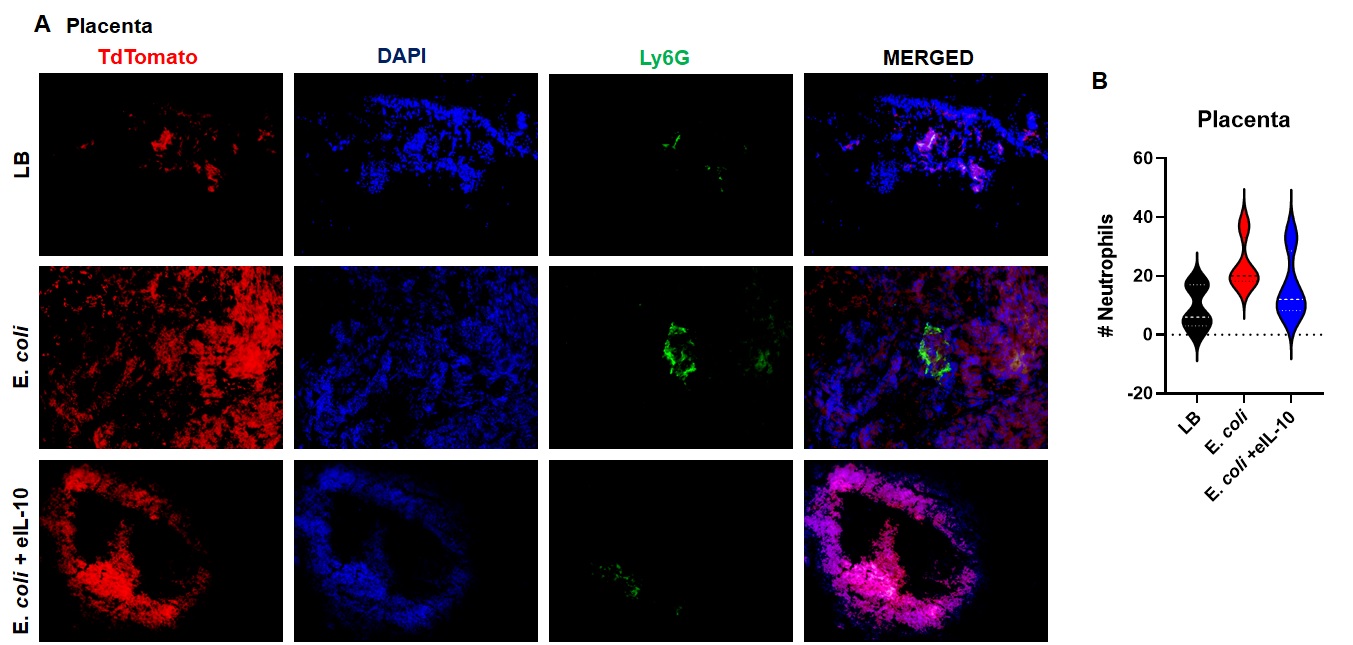

Supplement: Supplementary Figure 3 — (A). Immunohistochemistry of placenta 24 hours after different treatments (Control, E. coli, E. coli+eIL-10 and eIL-10). Tissues were stained for Ly6G (green) to show neutrophils and DAPI (blue) to show nuclei. (B). Violin graphs show significant increase in neutrophil levels after challenge with E. coli as seen in IHC fluorescent microscopy. (Control liquid broth in black, E. coli in red, E. coli+eIL-10 in blue and eIL-10 alone in pink) For all groups, n ≥ 5. Data are shown as means ± SEM. Data were analyzed using a one-way ANOVA with a Tukey’s post hoc test. *P ≤ 0.05, **P ≤ 0.01, ***P ≤ 0.001, ****P ≤ 0.0001. [file Image_3.jpeg]

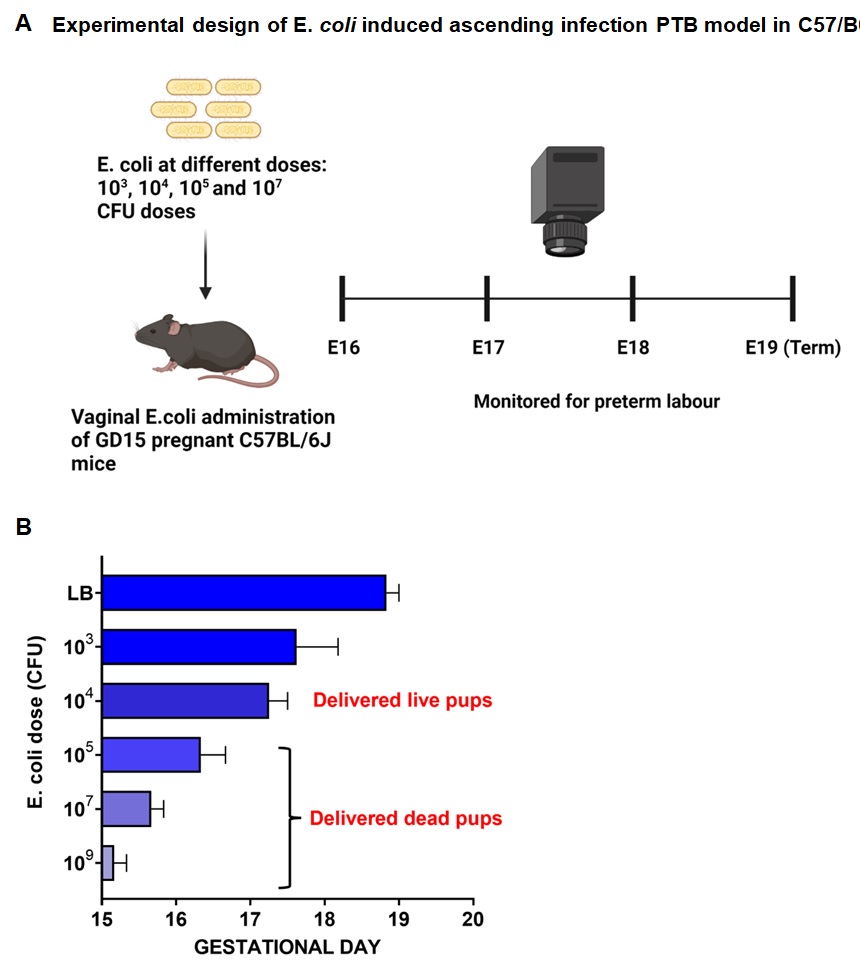

Supplement: Supplementary Figure 4 — (A). To determine dose of E. coli in ascending infection-induced PTB, on gestation day 15, pregnant C57BL/6J mice were challenged with different doses of E. coli though vaginal administration. PTB was monitored under video recording. (B). Compared to LB (liquid broth) control animals, vaginal administration of 109, 107 and 105 CFU E. coli resulted in PTB within 36 hours, but delivered dead pups. However, 104 CFU dose induced PTB within 48 hours and delivered PTB live pups which is clinically relevant model to humans. The 103 CFU group showed 50% PTB (2/4). For all groups, n ≥ 10. Data are shown as means ± SEM. Data were analyzed using a one-way ANOVA with a Tukey’s post hoc test. *P ≤ 0.05, **P ≤ 0.01, ***P ≤ 0.001, ****P ≤ 0.0001. [file Image_4.jpeg]

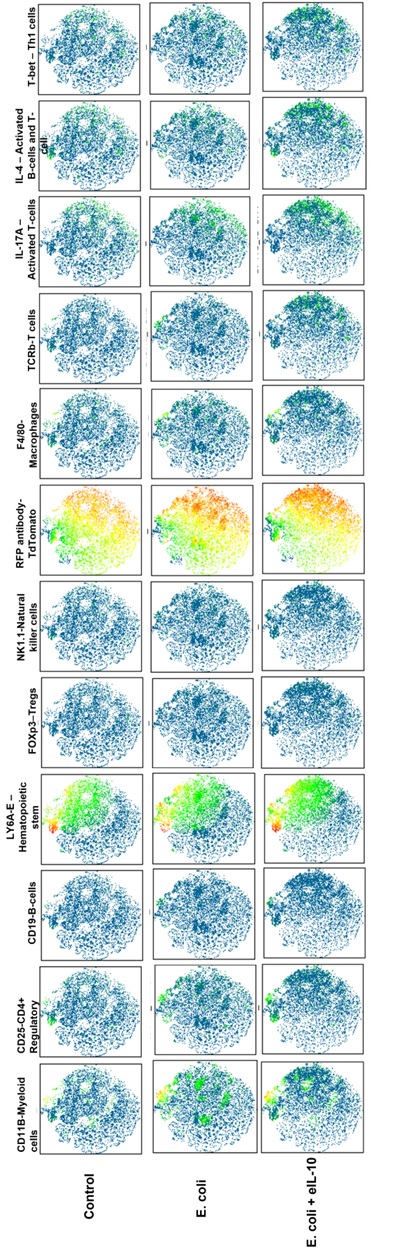

Supplement: Supplementary Figure 5 — Representative phenographs of individual markers among different groups of the FM tissues obtained by FlowJo Phenograph plug ins CyTOF data analysis. [file Image_5.jpeg]
