# Supplementary material for: Extracellular Vesicles-mediated recombinant IL-10 protects against ascending infection-associated preterm birth by reducing fetal inflammatory response
Source: Front Immunol. 2023 Aug 4;14:1196453. doi: 10.3389/fimmu.2023.1196453 (PMC10437065; doi:10.3389/fimmu.2023.1196453)
Supplement: Supplementary file 6 [file Table_1.pdf]

Table 1. Preterm delivery in CD-1 mice

Treatment	N	% PTB	hours to PTB	hours delayed
PBS	10	0 (0/10)	125.0±7.0	n/a
LPS	12	100 (12/12)	6.0±1.5	n/a
LPS+Naïve EVs	3	100 (3/3)	9.0 ± 3.4	n/a
LPS+eIL-10	8	48.2(5/8)	20.8 ± 7.8	14.8
LPS+rIL-10	3	66.6 (2/3)	60.0 ± 12.0	54.7
**Ascending Infection**				
LB	3	0 (0/3)	120±5.0	n/a
E. *coli*	10	100 (10/10)	13.5+3.2	n/a
E. *coli* + eIL-10	8	37.3 (3/8)	71.25±11.58	57.75
E. *coli* + Gentamicin	8	37.3 (3/8)	22.00±1.155	10.5
E. *coli* + eIL-10+ Gentamicin	6	66.6 (4/6)	47.75±14.30	34.25
eIL-10	5	0 (0/5)	123±6.5	n/a
E. *coli* + rIL-10	3	100 (3/3)	13.25±0.825	n/a
