# Supplementary material for: Extracellular Vesicles-mediated recombinant IL-10 protects against ascending infection-associated preterm birth by reducing fetal inflammatory response
Source: Front Immunol. 2023 Aug 4;14:1196453. doi: 10.3389/fimmu.2023.1196453 (PMC10437065; doi:10.3389/fimmu.2023.1196453)
Supplement: Supplementary file 7 [file Table_2.pdf]

Table 2. List of CyTOF conjugated antibodies panel

S.NO	TaggedAbs.description	Target	label	clone	Source
1	PU.1(Ms) 161Dy	PU.1	161Dy	7C2C34	BioLegend
2	NK1.1(Ms) 170Er	NK1.1, CD161b/c, Ly-55	170Er	PK136	DVS-Fluidigm
3	T-bet 154Sm	T-bet	154Sm	4B10	BioLegend
4	RORgt 163Dy	RORg(t)	163Dy	B2D	eBioscience
5	CD117(Ms) 169Tm	CD117	169Tm	2B8	BioLegend
6	CD25(Ms) 150Nd (MDA)	CD25	150Nd	3C7	BioLegend
7	IL-4(Ms) 166Er	IL-4	166Er	11B11	DVS-Fluidigm
8	CD45(Ms) 89Y	CD45(Ms)	89Y	30-F11	DVS-Fluidigm
9	IL-17A(Ms) 174Yb	IL-17A	174Yb	TC11-18H10.1	DVS-Fluidigm
10	Ly-6A/E 164Dy	Ly-6A/E, Sca-1	164Dy	D7	DVS-Fluidigm
11	CD19(Ms) 149Sm (MDA)	CD19	149Sm	4D5	BioLegend
12	CD4(Ms) 115In	CD4(Ms)	115In	RM4-5	BioLegend
13	CD8a(Ms) 146Nd (MDA)	CD8a	146Nd	53-6.7	BioLegend
14	TCRb(Ms) 143Nd	TCR _	143Nd	H57-597	DVS-Fluidigm
15	F4/80 159Tb (MDA)	F4/80	159Tb	BM8	BioLegend
16	CD11c(Ms) 142Nd	CD11c	142Nd	N418	DVS-Fluidigm
17	IFNg(Ms) 165Ho	IFNg	165Ho	XMG1.2	DVS-Fluidigm
18	Foxp3(Ms) 158Gd	Foxp3	158Gd	FJK-16s	DVS-Fluidigm
19	Siglec-F 172Yb	Siglec-F	172Yb	E50-2440	BD
20	CD14(Ms) 156Gd	CD14	156Gd	Sa14-2	DVS-Fluidigm
21	GATA3 145Nd	GATA3	145Nd	TWAJ	eBioscience
22	CD11b 148Nd	CD11b	148Nd	M1/70	DVS-Fluidigm
23	I-A/I-E 209Bi	I-A/I-E, MHC-II	209Bi	M5/114.15.2	DVS-Fluidigm
24	CD86 152sm	CD86	152Sm	In-house	BioLegend
25	TdTomato 172Lu	RFP	175Lu	In-house	BioLegend
