# Supplementary material for: Extracellular Vesicles-mediated recombinant IL-10 protects against ascending infection-associated preterm birth by reducing fetal inflammatory response
Source: Front Immunol. 2023 Aug 4;14:1196453. doi: 10.3389/fimmu.2023.1196453 (PMC10437065; doi:10.3389/fimmu.2023.1196453)
Supplement: Supplementary file 8 [file Table_3.docx]

**Supplement Table 3. Characterization of clusters form the CyTOF data**

| **S. No** | **Cluster profiling** | **Cluster characterization** | **References** |
| --- | --- | --- | --- |
| 1 | PU.1, RORγt, IL-4,T-bet,CD117 and IL-17A | Innate Lymphocyte cells | (Sahir et al. 2020; Triggianese et al. 2016; Withers et al. 2016) (Trabanelli, Gomez-Cadena, and Jandus 2019; Li et al. 2018) |
| 2 | CD11C, Ly6A-E, Siglec F | Neutrophils | (McGill, Lu, and Benayoun 2021; Ryu et al. 2022; Pfirschke et al. 2020) |
| 3 | CD19 | B-Lymphocytes | (Wang, Wei, and Liu 2012) |
| 4 | FOXP3 ROR CD25+ | T-regulatory cells | (Churlaud et al. 2015; Sakaguchi et al. 2010) |
| 5 | LY6A-E, NK1.1 | Natural Killer cells | (McFarland et al. 2021; Hirsh et al. 2004) |
| 6 | CD11B, Ly6A, CD45,1IL-17a | Myeloid-derived suppressor cells | (Bronte et al. 2016) |
| 7 | TCR, IL-17A | T-Helper cells | (Zhao et al. 2014) |
| 8 | TCR, RORγt, IL-17a | TH-17 cells | (Zhao et al. 2014; Chi et al. 2022; Capone and Volpe 2020) |
| 9 | CD11C+,MHCII+ | Conventional Dendritic cells | (Ueffing et al. 2017) |
| 10 | CD11B,CD117+ | Mast cells | (Sulce et al. 2018; Ye et al. 2015) |
| 11 | CD45+,Ly6A-E,CD14,F4/80+,IFN | Activated Macrophages | (Liu et al. 2020; Mily et al. 2020; Lee et al. 2018; Jablonski et al. 2015) |
| 12 | GATA,CD25, T-bet,CD117, SiglecF | Regulatory CD4+ T-cells | (Liechti and Roederer 2019; Mousset et al. 2019; Schmidt et al. 2016; Lu et al. 2011) |
| 13 | Ly6A-E, Tbet | Hemopoietic cells | (Whitmire, Eam, and Whitton 2009; Sahir et al. 2020) |

Bronte, V., S. Brandau, S. H. Chen, M. P. Colombo, A. B. Frey, T. F. Greten, S. Mandruzzato, P. J. Murray, A. Ochoa, S. Ostrand-Rosenberg, P. C. Rodriguez, A. Sica, V. Umansky, R. H. Vonderheide, and D. I. Gabrilovich. 2016. "Recommendations for myeloid-derived suppressor cell nomenclature and characterization standards." *Nat Commun* 7: 12150. <https://doi.org/10.1038/ncomms12150>. <https://www.ncbi.nlm.nih.gov/pubmed/27381735>.

Capone, A., and E. Volpe. 2020. "Transcriptional Regulators of T Helper 17 Cell Differentiation in Health and Autoimmune Diseases." *Front Immunol* 11: 348. <https://doi.org/10.3389/fimmu.2020.00348>. <https://www.ncbi.nlm.nih.gov/pubmed/32226427>.

Chi, X., W. Jin, X. Zhao, T. Xie, J. Shao, X. Bai, Y. Jiang, X. Wang, and C. Dong. 2022. "RORγt expression in mature T." *Sci Adv* 8 (34): eabn7774. <https://doi.org/10.1126/sciadv.abn7774>. <https://www.ncbi.nlm.nih.gov/pubmed/36026450>.

Churlaud, G., F. Pitoiset, F. Jebbawi, R. Lorenzon, B. Bellier, M. Rosenzwajg, and D. Klatzmann. 2015. "Human and Mouse CD8(+)CD25(+)FOXP3(+) Regulatory T Cells at Steady State and during Interleukin-2 Therapy." *Front Immunol* 6: 171. <https://doi.org/10.3389/fimmu.2015.00171>. <https://www.ncbi.nlm.nih.gov/pubmed/25926835>.

Hirsh, M., V. Kaplan, L. Dyugovskaya, and M. M. Krausz. 2004. "Response of lung NK1.1-positive natural killer cells to experimental sepsis in mice." *Shock* 22 (1): 40-5. <https://doi.org/10.1097/01.shk.0000129758.81361.45>. <https://www.ncbi.nlm.nih.gov/pubmed/15201700>.

Jablonski, K. A., S. A. Amici, L. M. Webb, J.e D Ruiz-Rosado, P. G. Popovich, S. Partida-Sanchez, and M. Guerau-de-Arellano. 2015. "Novel Markers to Delineate Murine M1 and M2 Macrophages." *PLoS One* 10 (12): e0145342. <https://doi.org/10.1371/journal.pone.0145342>. <https://www.ncbi.nlm.nih.gov/pubmed/26699615>.

Lee, S. A., S. Noel, M. Sadasivam, M. E. Allaf, P. M. Pierorazio, A. R. A. Hamad, and H. Rabb. 2018. "Characterization of kidney CD45intCD11bintF4/80+MHCII+CX3CR1+Ly6C- "intermediate mononuclear phagocytic cells"." *PLoS One* 13 (6): e0198608. <https://doi.org/10.1371/journal.pone.0198608>. <https://www.ncbi.nlm.nih.gov/pubmed/29856833>.

Li, N., V. van Unen, T. Höllt, A. Thompson, J. van Bergen, N. Pezzotti, E. Eisemann, A. Vilanova, S. M. Chuva de Sousa Lopes, B. P. F. Lelieveldt, and F. Koning. 2018. "Mass cytometry reveals innate lymphoid cell differentiation pathways in the human fetal intestine." *J Exp Med* 215 (5): 1383-1396. <https://doi.org/10.1084/jem.20171934>. <https://www.ncbi.nlm.nih.gov/pubmed/29511064>.

Liechti, T., and M. Roederer. 2019. "OMIP-060: 30-Parameter Flow Cytometry Panel to Assess T Cell Effector Functions and Regulatory T Cells." *Cytometry A* 95 (11): 1129-1134. <https://doi.org/10.1002/cyto.a.23853>. <https://www.ncbi.nlm.nih.gov/pubmed/31334913>.

Liu, Z., Y. Gu, A. Shin, S. Zhang, and F. Ginhoux. 2020. "Analysis of Myeloid Cells in Mouse Tissues with Flow Cytometry." *STAR Protoc* 1 (1): 100029. <https://doi.org/10.1016/j.xpro.2020.100029>. <https://www.ncbi.nlm.nih.gov/pubmed/33111080>.

Lu, Y., C. Malmhäll, M. Sjöstrand, M. Rådinger, S. E. O'Neil, J. Lötvall, and A. Bossios. 2011. "Expansion of CD4(+) CD25(+) and CD25(-) T-Bet, GATA-3, Foxp3 and RORγt cells in allergic inflammation, local lung distribution and chemokine gene expression." *PLoS One* 6 (5): e19889. <https://doi.org/10.1371/journal.pone.0019889>. <https://www.ncbi.nlm.nih.gov/pubmed/21625544>.

McFarland, A. P., A. Yalin, S. Y. Wang, V. S. Cortez, T. Landsberger, R. Sudan, V. Peng, H. L. Miller, B. Ricci, E. David, R. Faccio, I. Amit, and M. Colonna. 2021. "Multi-tissue single-cell analysis deconstructs the complex programs of mouse natural killer and type 1 innate lymphoid cells in tissues and circulation." *Immunity* 54 (6): 1320-1337.e4. <https://doi.org/10.1016/j.immuni.2021.03.024>. <https://www.ncbi.nlm.nih.gov/pubmed/33945787>.

McGill, C. J., R. J. Lu, and B. A. Benayoun. 2021. "Protocol for analysis of mouse neutrophil NETosis by flow cytometry." *STAR Protoc* 2 (4): 100948. <https://doi.org/10.1016/j.xpro.2021.100948>. <https://www.ncbi.nlm.nih.gov/pubmed/34820637>.

Mily, A., S. Kalsum, M. G. Loreti, R. S. Rekha, J. R. Muvva, M. Lourda, and S. Brighenti. 2020. "Polarization of M1 and M2 Human Monocyte-Derived Cells and Analysis with Flow Cytometry upon Mycobacterium tuberculosis Infection." *J Vis Exp* (163). <https://doi.org/10.3791/61807>. <https://www.ncbi.nlm.nih.gov/pubmed/33016941>.

Mousset, C. M., W. Hobo, R. Woestenenk, F. Preijers, H. Dolstra, and A. B. van der Waart. 2019. "Comprehensive Phenotyping of T Cells Using Flow Cytometry." *Cytometry A* 95 (6): 647-654. <https://doi.org/10.1002/cyto.a.23724>. <https://www.ncbi.nlm.nih.gov/pubmed/30714682>.

Pfirschke, C., C. Engblom, J. Gungabeesoon, Y. Lin, S. Rickelt, R. Zilionis, M. Messemaker, M. Siwicki, G. M. Gerhard, A. Kohl, E. Meylan, R. Weissleder, A. M. Klein, and M. J. Pittet. 2020. "Tumor-Promoting Ly-6G." *Cell Rep* 32 (12): 108164. <https://doi.org/10.1016/j.celrep.2020.108164>. <https://www.ncbi.nlm.nih.gov/pubmed/32966785>.

Ryu, S., J. W. Shin, S. Kwon, J. Lee, Y. C. Kim, Y. S. Bae, D. K. Kim, Y. S. Kim, S. H. Yang, and H. Y. Kim. 2022. "Siglec-F-expressing neutrophils are essential for creating a profibrotic microenvironment in renal fibrosis." *J Clin Invest* 132 (12). <https://doi.org/10.1172/JCI156876>. <https://www.ncbi.nlm.nih.gov/pubmed/35482420>.

Sahir, F., J. M. Mateo, M. Steinhoff, and K. S. Siveen. 2020. "Development of a 43 color panel for the characterization of conventional and unconventional T-cell subsets, B cells, NK cells, monocytes, dendritic cells, and innate lymphoid cells using spectral flow cytometry." *Cytometry A*. <https://doi.org/10.1002/cyto.a.24288>. <https://www.ncbi.nlm.nih.gov/pubmed/33336868>.

Sakaguchi, S., M. Miyara, C. M. Costantino, and D. A. Hafler. 2010. "FOXP3+ regulatory T cells in the human immune system." *Nat Rev Immunol* 10 (7): 490-500. <https://doi.org/10.1038/nri2785>. <https://www.ncbi.nlm.nih.gov/pubmed/20559327>.

Schmidt, A., S. Éliás, R. N. Joshi, and J. Tegnér. 2016. "In Vitro Differentiation of Human CD4+FOXP3+ Induced Regulatory T Cells (iTregs) from Naïve CD4+ T Cells Using a TGF-β-containing Protocol." *J Vis Exp* (118). <https://doi.org/10.3791/55015>. <https://www.ncbi.nlm.nih.gov/pubmed/28060341>.

Sulce, M., L. Marconato, M. Martano, S. Iussich, A. Dentini, M. Melega, B. Miniscalco, and F. Riondato. 2018. "Utility of flow cytometry in canine primary cutaneous and matched nodal mast cell tumor." *Vet J* 242: 15-23. <https://doi.org/10.1016/j.tvjl.2018.10.004>. <https://www.ncbi.nlm.nih.gov/pubmed/30503539>.

Trabanelli, S., A. Gomez-Cadena, and C. Jandus. 2019. "Immunophenotyping of Human Innate Lymphoid Cells." *Methods Mol Biol* 2032: 179-192. <https://doi.org/10.1007/978-1-4939-9650-6_10>. <https://www.ncbi.nlm.nih.gov/pubmed/31522419>.

Triggianese, P., P. Conigliaro, M. S. Chimenti, L. Biancone, G. Monteleone, R. Perricone, and I. Monteleone. 2016. "Evidence of IL-17 producing innate lymphoid cells in peripheral blood from patients with enteropathic spondyloarthritis." *Clin Exp Rheumatol* 34 (6): 1085-1093. <https://www.ncbi.nlm.nih.gov/pubmed/27782868>.

Ueffing, K., H. Abberger, A. M. Westendorf, K. Matuschewski, J. Buer, and W. Hansen. 2017. "Conventional CD11c." *Front Immunol* 8: 1333. <https://doi.org/10.3389/fimmu.2017.01333>. <https://www.ncbi.nlm.nih.gov/pubmed/29085373>.

Wang, K., G. Wei, and D. Liu. 2012. "CD19: a biomarker for B cell development, lymphoma diagnosis and therapy." *Exp Hematol Oncol* 1 (1): 36. <https://doi.org/10.1186/2162-3619-1-36>. <https://www.ncbi.nlm.nih.gov/pubmed/23210908>.

Whitmire, J. K., B. Eam, and J. L. Whitton. 2009. "Mice deficient in stem cell antigen-1 (Sca1, Ly-6A/E) develop normal primary and memory CD4+ and CD8+ T-cell responses to virus infection." *Eur J Immunol* 39 (6): 1494-504. <https://doi.org/10.1002/eji.200838959>. <https://www.ncbi.nlm.nih.gov/pubmed/19384870>.

Withers, D. R., M. R. Hepworth, X. Wang, E. C. Mackley, E. E. Halford, E. E. Dutton, C. L. Marriott, V. Brucklacher-Waldert, M. Veldhoen, J. Kelsen, R. N. Baldassano, and G. F. Sonnenberg. 2016. "Transient inhibition of ROR-γt therapeutically limits intestinal inflammation by reducing TH17 cells and preserving group 3 innate lymphoid cells." *Nat Med* 22 (3): 319-23. <https://doi.org/10.1038/nm.4046>. <https://www.ncbi.nlm.nih.gov/pubmed/26878233>.

Ye, J. X., Y. Liu, Y. Qin, X. L. Ma, H. H. Zhong, Y. Zhang, and X. Y. Shi. 2015. "Mast cells or not? - CD117 positive cells in esophageal leiomyoma." *Histol Histopathol* 30 (5): 581-8. <https://doi.org/10.14670/HH-30.581>. <https://www.ncbi.nlm.nih.gov/pubmed/25487836>.

Zhao, L., Y. Chou, Y. Jiang, Z. Jiang, and C. Q. Chu. 2014. "Analysis of IL-17 production by flow cytometry and ELISPOT assays." *Methods Mol Biol* 1172: 243-56. <https://doi.org/10.1007/978-1-4939-0928-5_22>. <https://www.ncbi.nlm.nih.gov/pubmed/24908311>.
